# Relevance of genetic testing in the gene-targeted trial era: the Rostock Parkinson’s disease study

**DOI:** 10.1093/brain/awae188

**Published:** 2024-08-01

**Authors:** Ana Westenberger, Volha Skrahina, Tatiana Usnich, Christian Beetz, Eva-Juliane Vollstedt, Björn-Hergen Laabs, Jefri J Paul, Filipa Curado, Snezana Skobalj, Hanaa Gaber, Maria Olmedillas, Xenia Bogdanovic, Najim Ameziane, Nathalie Schell, Jan Olav Aasly, Mitra Afshari, Pinky Agarwal, Jason Aldred, Fernando Alonso-Frech, Roderick Anderson, Rui Araújo, David Arkadir, Micol Avenali, Mehmet Balal, Sandra Benizri, Sagari Bette, Perminder Bhatia, Michael Bonello, Pedro Braga-Neto, Sarah Brauneis, Francisco Eduardo Costa Cardoso, Francesco Cavallieri, Joseph Classen, Lisa Cohen, Della Coletta, David Crosiers, Paskal Cullufi, Khashayar Dashtipour, Meltem Demirkiran, Patricia de Carvalho Aguiar, Anna De Rosa, Ruth Djaldetti, Okan Dogu, Maria Gabriela dos Santos Ghilardi, Carsten Eggers, Bulent Elibol, Aaron Ellenbogen, Sibel Ertan, Giorgio Fabiani, Björn H Falkenburger, Simon Farrow, Tsviya Fay-Karmon, Gerald J Ferencz, Erich Talamoni Fonoff, Yara Dadalti Fragoso, Gençer Genç, Arantza Gorospe, Francisco Grandas, Doreen Gruber, Mark Gudesblatt, Tanya Gurevich, Johann Hagenah, Hasmet A Hanagasi, Sharon Hassin-Baer, Robert A Hauser, Jorge Hernández-Vara, Birgit Herting, Vanessa K Hinson, Elliot Hogg, Michele T Hu, Eduardo Hummelgen, Kelly Hussey, Jon Infante, Stuart H Isaacson, Serge Jauma, Natalia Koleva-Alazeh, Gregor Kuhlenbäumer, Andrea Kühn, Irene Litvan, Lydia López-Manzanares, McKenzie Luxmore, Sujeena Manandhar, Veronique Marcaud, Katerina Markopoulou, Connie Marras, Mark McKenzie, Michele Matarazzo, Marcelo Merello, Brit Mollenhauer, John C Morgan, Stephen Mullin, Thomas Musacchio, Bennett Myers, Anna Negrotti, Anette Nieves, Zeev Nitsan, Nader Oskooilar, Özgür Öztop-Çakmak, Gian Pal, Nicola Pavese, Antonio Percesepe, Tommaso Piccoli, Carolina Pinto de Souza, Tino Prell, Mark Pulera, Jason Raw, Kathrin Reetz, Johnathan Reiner, David Rosenberg, Marta Ruiz-Lopez, Javier Ruiz Martinez, Esther Sammler, Bruno Lopes Santos-Lobato, Rachel Saunders-Pullman, Ilana Schlesinger, Christine M Schofield, Artur F Schumacher-Schuh, Burton Scott, Ángel Sesar, Stuart J Shafer, Ray Sheridan, Monty Silverdale, Rani Sophia, Mariana Spitz, Pantelis Stathis, Fabrizio Stocchi, Michele Tagliati, Yen F Tai, Annelies Terwecoren, Sven Thonke, Lars Tönges, Giulia Toschi, Vitor Tumas, Peter Paul Urban, Laura Vacca, Wim Vandenberghe, Enza Maria Valente, Franco Valzania, Lydia Vela-Desojo, Caroline Weill, David Weise, Joanne Wojcieszek, Martin Wolz, Gilad Yahalom, Gul Yalcin-Cakmakli, Simone Zittel, Yair Zlotnik, Krishna K Kandaswamy, Alexander Balck, Henrike Hanssen, Max Borsche, Lara M Lange, Ilona Csoti, Katja Lohmann, Meike Kasten, Norbert Brüggemann, Arndt Rolfs, Christine Klein, Peter Bauer

**Affiliations:** Institute of Neurogenetics, University of Lübeck, University Medical Center Schleswig-Holstein, 23538 Lübeck, Schleswig-Holstein, Germany; CENTOGENE GmbH, 18055 Rostock, Mecklenburg-Vorpommern, Germany; Institute of Neurogenetics, University of Lübeck, University Medical Center Schleswig-Holstein, 23538 Lübeck, Schleswig-Holstein, Germany; CENTOGENE GmbH, 18055 Rostock, Mecklenburg-Vorpommern, Germany; Institute of Neurogenetics, University of Lübeck, University Medical Center Schleswig-Holstein, 23538 Lübeck, Schleswig-Holstein, Germany; Institute of Medical Biometry and Statistics, University of Lübeck, University Medical Center Schleswig-Holstein, 23562 Lübeck, Schleswig-Holstein, Germany; CENTOGENE GmbH, 18055 Rostock, Mecklenburg-Vorpommern, Germany; CENTOGENE GmbH, 18055 Rostock, Mecklenburg-Vorpommern, Germany; CENTOGENE GmbH, 18055 Rostock, Mecklenburg-Vorpommern, Germany; CENTOGENE GmbH, 18055 Rostock, Mecklenburg-Vorpommern, Germany; Department of Clinical Project Management, IQVIA, 60549 Frankfurt am Main, Hessen, Germany; CENTOGENE GmbH, 18055 Rostock, Mecklenburg-Vorpommern, Germany; CENTOGENE GmbH, 18055 Rostock, Mecklenburg-Vorpommern, Germany; CENTOGENE GmbH, 18055 Rostock, Mecklenburg-Vorpommern, Germany; Institute of Neurogenetics, University of Lübeck, University Medical Center Schleswig-Holstein, 23538 Lübeck, Schleswig-Holstein, Germany; Department of Neurology, St. Olavs Hospital, 7006 Trondheim, Trøndelag, Norway; Department of Neuroscience, Norwegian University of Science and Technology, 7034 Trondheim, Norway; Department of Neurological Sciences, Rush University Medical Center, Chicago, IL 60612, USA; Evergreen Health Neuroscience Institute, Kirkland, WA 98034, USA; Inland Northwest Research, Spokane, WA 99202, USA; Department of Neurology, Movement Disorders Unit, Hospital Clínico San Carlos, 28040 Madrid, Madrid, Spain; Tucson Neuroscience Research, Tucson, AZ 85710, USA; Department of Neurology, Centro Hospitalar Universitário de São João, 4200-319 Porto, Porto District, Portugal; Department of Clinical Neurosciences and Mental Health, Faculty of Medicine, University of Porto, 4200-319 Porto, Porto District, Portugal; Department of Neurology, Faculty of Medicine, Hadassah Medical Organization, Hebrew University, 91120 Jerusalem, Jerusalem District, Israel; Neurogenetics Research Center, IRCCS Mondino Foundation, 27100 Pavia, Italy; Department of Brain and Behavioral Sciences, University of Pavia, 27100 Pavia, Lombardy, Italy; Department of Neurology, School of Medicine, Çukurova University, 01330 Adana, Adana, Turkey; Movement Disorders Unit, Assuta Ramat Ha Hayal Hospital, 69710 Tel Aviv, Tel Aviv District, Israel; Parkinson’s Disease and Movement Disorders Center of Boca Raton, Boca Raton, FL 33486, USA; Neuro Pain Medical Center, Fresno, CA 93720, USA; Department of Neurology, The Walton Centre NHS Foundation Trust, Liverpool, Merseyside L9 7LJ, UK; Division of Neurology, Department of Clinical Medicine, Federal University of Ceará, 60430-140 Fortaleza, Brazil; Center of Health Science, Universidade Estadual do Ceará, 60714-903 Fortaleza, Ceará, Brazil; Inland Northwest Research, Spokane, WA 99202, USA; Movement Disorders Unit, Neurology Service, Department of Internal Medicine, Federal University of Minas Gerais, 31270-901 Belo Horizonte, Minas Gerais, Brazil; Neurology Unit, Neuromotor and Rehabilitation Department, Azienda Unità Sanitaria Locale-IRCCS di Reggio Emilia, 42122 Reggio Emilia, Emilia-Romagna, Italy; Department of Neurology, Leipzig University Medical Center, 04103 Leipzig, Saxony, Germany; Wake Research, Raleigh, NC 27612, USA; Department of Neurology, Universidade do Estado do Amazonas, 69050-010 Manaus AM, Amazonas, Brazil; Department of Neurology, Antwerp University Hospital, 2650 Edegem, Flemish, Belgium; Translational Neurosciences, Faculty of Medicine and Health Sciences, University of Antwerp, 2610 Antwerp, Flemish, Belgium; Pediatric Department, University Hospital ‘Mother Teresa’, 1001 Tirana, Tirana County, Albania; Department of Neurology, Division of Movement Disorders, Loma Linda University School of Medicine, Loma Linda, CA 92354, USA; Department of Neurology, School of Medicine, Çukurova University, 01330 Adana, Adana, Turkey; Department of Neurology and Neurosurgery, Hospital Israelita Albert Einstein, 05651-901 Sao Paulo, Sao Paulo, Brazil; Department of Neurosciences and Reproductive and Odontostomatological Sciences, Federico II University, 80131 Naples, Campania Region, Italy; Department of Neurology, Movement Disorders Clinic, Rabin Medical Center-Beilinson Hospital, 49100 Petach Tikva, Central District, Israel; Sackler Faculty of Medicine, Tel Aviv University, 6997801 Tel Aviv, Tel Aviv District, Israel; Department of Neurology, Mersin University, 33343 Mersin, Mersin Province, Turkey; Laboratory of Neuroscience, Hospital Sírio-Libanês, 01308-050 São Paulo, São Paulo, Brazil; Department of Neurology, University of São Paulo Medical School, 01246-903 São Paulo, São Paulo, Brazil; Department of Neurology, University Hospital Marburg, 35037 Marburg, Hesse, Germany; Department of Neurology, Knappschaftskrankenhaus Bottrop, 46242 Bottrop, North Rhine-Westphalia, Germany; Department of Neurology, Faculty of Medicine, Hacettepe University, 06100 Ankara, Ankara, Turkey; Michigan Institute for Neurological Disorders, Farmington Hills, MI 48334, USA; Quest Research Institute, Farmington Hills, MI 48334, USA; Department of Neurology, Koç University, 34450 Istanbul, Istanbul, Turkey; Movement Disorders Unit, Hospital Angelina Caron, 83430-000 Curitiba, Paraná, Brazil; Department of Neurology, University Hospital and Faculty of Medicine Carl Gustav Carus, 01307 Dresden, Saxony, Germany; Clinical Research Center of Nevada, Las Vegas, NV 89119, USA; Sackler Faculty of Medicine, Tel Aviv University, 6997801 Tel Aviv, Tel Aviv District, Israel; Movement Disorders Institute and Department of Neurology, Chaim Sheba Medical Center, 52621 Ramat-Gan, Tel Aviv District, Israel; Shore Neurology, RWJBarnabas Health Medical Group, Toms River, NJ 08755, USA; Laboratory of Neuroscience, Hospital Sírio-Libanês, 01308-050 São Paulo, São Paulo, Brazil; Department of Neurology, University of São Paulo Medical School, 01246-903 São Paulo, São Paulo, Brazil; Department of Neurology, Universidade Metropolitana de Santos, 11070-100 Santos SP, São Paulo, Brazil; Department of Neurology, Şişli Etfal Training and Research Hospital, University of Health Sciences, 34371 Istanbul, Istanbul, Turkey; Department of Neurology, de Navarra University Hospital, 31008 Pamplona, Navarre, Spain; Movement Disorders Unit, University General Hospital Gregorio Marañón, 28007 Madrid, Madrid, Spain; Movement Disorders Clinic, 14547 Beelitz-Heilstätten, Brandenburg, Germany; NYU Langone South Shore Neurologic Associates, Islip, NY 11751, USA; Movement Disorders Unit, Neurological Institute, Tel Aviv Sourasky Medical Center, Tel Aviv University, 6423906 Tel Aviv, Tel Aviv District, Israel; Department of Neurology, Westküstenklinikum Heide, 25746 Heide, Schleswig-Holstein, Germany; Behavioral Neurology and Movement Disorders Unit, Department of Neurology, Istanbul Faculty of Medicine, Istanbul University, 34093 Istanbul, Istanbul, Turkey; Sackler Faculty of Medicine, Tel Aviv University, 6997801 Tel Aviv, Tel Aviv District, Israel; Movement Disorders Institute and Department of Neurology, Chaim Sheba Medical Center, 52621 Ramat-Gan, Tel Aviv District, Israel; University of South Florida Parkinson’s Disease and Movement Disorders Center of Excellence, Tampa, FL 33612, USA; Neurology Department, Vall d’Hebron University Hospital, Universitat Autònoma de Barcelona, 08035 Barcelona, Catalonia, Spain; Neurological Clinic, Diakonie-Klinikum Schwäbisch Hall, 74523 Schwäbisch Hall, Baden-Württemberg, Germany; Department of Neurology, Medical University of South Carolina, Charleston, SC 29425, USA; Department of Neurosurgery, Cedars-Sinai Medical Center, Movement Disorder Program, Los Angeles, CA 90048, USA; Nuffield Department of Clinical Neurosciences, Division of Clinical Neurology, University of Oxford, Oxford OX3 9DU, UK; Neurology Service, Hospital Angelina Caron, 83430-000 Curitiba, Paraná, Brazil; University of South Florida Parkinson’s Disease and Movement Disorders Center of Excellence, Tampa, FL 33612, USA; Service of Neurology, University Hospital ‘Marqués de Valdecilla (IDIVAL)’, University of Cantabria, and ‘Centro de Investigación Biomédica en Red de Enfermedades Neurodegenerativas (CIBERNED)’, 39008 Santander, Cantabria, Spain; Parkinson’s Disease and Movement Disorders Center of Boca Raton, Boca Raton, FL 33486, USA; Neurology Service, Hospital Universitari de Bellvitge, 08907 Barcelona, Catalonia, Spain; Parkinson-Center, Gertrudisklinik Biskirchen, 35638 Leun, Hesse, Germany; Department of Neurology, University Medical Center Schleswig-Holstein, Campus Kiel, 24105 Kiel, Schleswig-Holstein, Germany; Movement Disorder and Neuromodulation Unit, Department of Neurology, Charité, University Medicine Berlin, 10117 Berlin, Berlin, Germany; Parkinson and Other Movement Disorders Center, University of California San Diego Health, La Jolla, San Diego, CA 92037, USA; Department of Neurology, Movement Disorders Unit, La Princesa University Hospital, 28006 Madrid, Madrid, Spain; Department of Neurology, Duke University School of Medicine, Durham, NC 27710, USA; Evergreen Health Neuroscience Institute, Kirkland, WA 98034, USA; Department of Neurology, Saint Joseph Hospital, 75014 Paris, Île-de-France, France; Department of Neurology, NorthShore University HealthSystem, Evanston, IL 60201, USA; Department of Neurology, Pritzker School of Medicine, University of Chicago, Chicago, IL 60637, USA; The Edmond J Safra Program in Parkinson’s Disease, Toronto Western Hospital, University of Toronto, Toronto, Ontario M5T 2S8, Canada; ClinSearch, Chattanooga, TN 37421, USA; HM CINAC (Centro Integral de Neurociencias Abarca Campal), Fundación Hospitales de Madrid, Hospital Universitario HM Puerta del Sur, HM Hospitales, 28938 Madrid, Madrid, Spain; Movement Disorders Service FLENI, CONICET, C1428 Buenos Aires, Ciudad Autónoma de Buenos Aires (CABA), Argentina; Paracelsus-Elena-Klinik, 34128 Kassel, Hesse, Germany; Department of Neurology, University Medical Centre Göttingen, 37075 Göttingen, Lower Saxony, Germany; Movement & Memory Disorder Programs, Department of Neurology, Augusta University Medical Center, Augusta, GA 30912, USA; Institute of Translational and Stratified Medicine, University of Plymouth School of Medicine, Plymouth, Devon PL6 8BU, UK; Department of Neurology, University Hospital of Würzburg, 97080 Würzburg, Bavaria, Germany; DENT Neurologic Institute, Buffalo, NY 14226, USA; Department of General and Specialized Medicine, Neurology Unit, University Hospital of Parma, 43126 Parma, Emilia-Romagna, Italy; Renstar Medical Research, Ocala, FL 34471, USA; Department of Neurology, Barzilai Medical Center, 78278 Ashkelon, Southern District, Israel; Faculty of Health Sciences, Ben Gurion University of the Negev, 84105 Beer-Sheva, Southern District, Israel; Pharmacology Research Institute, Newport Beach, CA 92660, USA; Department of Neurology, Koç University, 34450 Istanbul, Istanbul, Turkey; Department of Neurology, Rutgers Robert Wood Johnson Medical School, New Brunswick, NJ 08901, USA; Clinical Ageing Research Unit, Newcastle University, Newcastle Upon Tyne, Tyne and Wear NE4 5PL, UK; Department of Medicine and Surgery, University of Parma, 43126 Parma, Emilia-Romagna, Italy; Unit of Neurology, Department of Biomedicine, Neurosciences and advanced Diagnostics (BiND), University of Palermo, 90127 Palermo, Sicily, Italy; Department of Neurology, São Francisco Hospital, University of São Paulo, 01236-030 São Paulo, São Paulo, Brazil; Department of Neurology, Jena University Hospital, 07747 Jena, Thuringia, Germany; Department of Geriatrics, Halle University Hospital, 06120 Halle, Saxony-Anhalt, Germany; Pharmacology Research Institute, Encino, CA 91316, USA; Clinical Research Unit, Pennine Acute Hospitals NHS Trust, Oldham, Greater Manchester OL1 2JH, UK; Department of Neurology, RWTH Aachen University, 52074 Aachen, North Rhine-Westphalia, Germany; JARA-BRAIN Institute Molecular Neuroscience and Neuroimaging, Research Centre Jülich, 52428 Jülich, North Rhine-Westphalia, Germany; Department of Neurology, Movement Disorders Clinic, Rabin Medical Center-Beilinson Hospital, 49100 Petach Tikva, Central District, Israel; Sackler Faculty of Medicine, Tel Aviv University, 6997801 Tel Aviv, Tel Aviv District, Israel; Pharmacology Research Institute, Los Alamitos, CA 90720, USA; Department of Neurology, University Hospital Cruces, Biocruces Research Institute, 48903 Barakaldo, Basque Country, Spain; Department of Neurology, Hospital Universitario Donostia, 20014 San Sebastian, Basque Country, Spain; Medical Research Council Protein Phosphorylation and Ubiquitylation Unit, University of Dundee, Dundee DD1 5EH, UK; Molecular and Clinical Medicine, Ninewells Hospital and Medical School, University of Dundee, Dundee DD1 9SY, UK; Department of Neurology, Hospital Ophir Loyola, 66050-380 Belem, Pará, Brazil; Department of Neurology, Icahn School of Medicine at Mount Sinai, New York, NY 10003, USA; Rambam Health Care Campus, Technion Faculty of Medicine, 31096 Haifa, Haifa District, Israel; Research and Development Unit, Royal Cornwall Hospitals Trust, Truro, Cornwall TR1 3LJ, UK; Neurological Services, Clinical Hospital of Porto Alegre, 90035-903 Porto Alegre, Rio Grande do Sul, Brazil; Department of Neurology, Duke University School of Medicine, Durham, NC 27710, USA; Department of Neurology, University Hospital of Santiago de Compostela, 15706 Santiago de Compostela, Galicia, Spain; Vero Beach Neurology and Research Institute, Vero Beach, FL 32960, USA; Geriatric Medicine, Royal Devon and Exeter Hospital NHS Foundation Trust, Exeter, Devon EX2 5DW, UK; Division of Neurology, Salford Royal NHS Foundation Trust, Manchester Academic Health Science Centre, University of Manchester, Manchester, Greater Manchester M6 8HD, UK; Department of Geriatric Medicine, Yeovil Hospital, Yeovil, Somerset BA21 4AT, UK; Neurology, Pedro Ernesto University Hospital, 20551-030 Rio de Janeiro, Rio de Janeiro, Brazil; Department of Neurology, Mediterraneo Hospital, 166 75 Glyfada-Athens, Attica, Greece; University and Institute for Research and Medical Care, IRCCS San Raffaele, 00166 Rome, Lazio, Italy; Nuffield Department of Clinical Neurosciences, Division of Clinical Neurology, University of Oxford, Oxford OX3 9DU, UK; Division of Medicine and Integrated Care, Charing Cross Hospital, Imperial College Healthcare Trust, London W6 8RF, UK; Department of Neurology, Damiaan Hospital, 8400 Ostend, Flanders, Belgium; Department of Neurology, Klinikum Hanau, 63450 Hanau, Hesse, Germany; Department of Neurology, St. Josef-Hospital and Neurodegeneration Research, Protein Research Unit Ruhr (PURE), Ruhr University Bochum, 44791 Bochum, North Rhine-Westphalia, Germany; Neurodegeneration Research, Protein Research Unit Ruhr (PURE), Ruhr University Bochum, 44791 Bochum, North Rhine-Westphalia, Germany; Neurology Unit, Neuromotor and Rehabilitation Department, Azienda Unità Sanitaria Locale-IRCCS di Reggio Emilia, 42122 Reggio Emilia, Emilia-Romagna, Italy; Department of Neurosciences and Behavioral Sciences, Ribeirao Preto Medical School of University of São Paulo, 14049-900 São Paulo, São Paulo, Brazil; Department of Neurology, Asklepios Klinik Barmbek, 22307 Hamburg, Hamburg, Germany; University and Institute for Research and Medical Care, IRCCS San Raffaele, 00166 Rome, Lazio, Italy; Department of Neurology, University Hospitals Leuven, 3000 Leuven, Flanders, Belgium; Department of Neurosciences, KU Leuven, 3000 Leuven, Flanders, Belgium; Neurogenetics Research Center, IRCCS Mondino Foundation, 27100 Pavia, Italy; Department of Molecular Medicine, University of Pavia, 27100 Pavia, Lombardy, Italy; Neurology Unit, Neuromotor and Rehabilitation Department, Azienda Unità Sanitaria Locale-IRCCS di Reggio Emilia, 42122 Reggio Emilia, Emilia-Romagna, Italy; Neurology Unit, Hospital Fundación Alcorcón, 28922 Madrid, Madrid, Spain; Neurogenetics Research Center, IRCCS Mondino Foundation, 27100 Pavia, Italy; Department of Neurology, Asklepios Fachklinikum Stadtroda, 07646 Stadtroda, Thuringia, Germany; Department of Neurology, University of Leipzig, 04103 Leipzig, Saxony, Germany; School of Medicine, Indiana University, Indianapolis, IN 46202, USA; Department of Neurology, Elblandklinikum Meißen, 01662 Meißen, Saxony, Germany; Department of Neurology and the Movement Disorders Unit, Shaare Zedek Medical Center, 9103102 Jerusalem, Jerusalem District, Israel; Department of Neurology, Faculty of Medicine, Hacettepe University, 06100 Ankara, Ankara, Turkey; Department of Neurology, University Medical Center Hamburg-Eppendorf, 20246 Hamburg, Hamburg, Germany; Neurology Department, Soroka University Medical Center, 84101 Beer Sheva, Southern District, Israel; CENTOGENE GmbH, 18055 Rostock, Mecklenburg-Vorpommern, Germany; Institute of Neurogenetics, University of Lübeck, University Medical Center Schleswig-Holstein, 23538 Lübeck, Schleswig-Holstein, Germany; Department of Neurology, University of Lübeck, 23562 Lübeck, Schleswig-Holstein, Germany; Institute of Neurogenetics, University of Lübeck, University Medical Center Schleswig-Holstein, 23538 Lübeck, Schleswig-Holstein, Germany; Department of Neurology, University of Lübeck, 23562 Lübeck, Schleswig-Holstein, Germany; Institute of Neurogenetics, University of Lübeck, University Medical Center Schleswig-Holstein, 23538 Lübeck, Schleswig-Holstein, Germany; Department of Neurology, University of Lübeck, 23562 Lübeck, Schleswig-Holstein, Germany; Institute of Neurogenetics, University of Lübeck, University Medical Center Schleswig-Holstein, 23538 Lübeck, Schleswig-Holstein, Germany; Department of Neurology, University of Lübeck, 23562 Lübeck, Schleswig-Holstein, Germany; Neurology Service, Hospital Universitari de Bellvitge, 08907 Barcelona, Catalonia, Spain; Institute of Neurogenetics, University of Lübeck, University Medical Center Schleswig-Holstein, 23538 Lübeck, Schleswig-Holstein, Germany; Institute of Neurogenetics, University of Lübeck, University Medical Center Schleswig-Holstein, 23538 Lübeck, Schleswig-Holstein, Germany; Institute of Neurogenetics, University of Lübeck, University Medical Center Schleswig-Holstein, 23538 Lübeck, Schleswig-Holstein, Germany; Department of Neurology, University of Lübeck, 23562 Lübeck, Schleswig-Holstein, Germany; CENTOGENE GmbH, 18055 Rostock, Mecklenburg-Vorpommern, Germany; Department of Neurology, University of Rostock, 18057 Rostock, Mecklenburg-Vorpommern, Germany; Institute of Neurogenetics, University of Lübeck, University Medical Center Schleswig-Holstein, 23538 Lübeck, Schleswig-Holstein, Germany; CENTOGENE GmbH, 18055 Rostock, Mecklenburg-Vorpommern, Germany; Department of Internal Medicine, University of Rostock, 18057 Rostock, Mecklenburg-Vorpommern, Germany

**Keywords:** Parkinson’s disease, genetic factors, genetic testing, next-generation sequencing, *LRRK2*, *GBA1*

## Abstract

Estimates of the spectrum and frequency of pathogenic variants in Parkinson’s disease (PD) in different populations are currently limited and biased. Furthermore, although therapeutic modification of several genetic targets has reached the clinical trial stage, a major obstacle in conducting these trials is that PD patients are largely unaware of their genetic status and, therefore, cannot be recruited. Expanding the number of investigated PD-related genes and including genes related to disorders with overlapping clinical features in large, well-phenotyped PD patient groups is a prerequisite for capturing the full variant spectrum underlying PD and for stratifying and prioritizing patients for gene-targeted clinical trials. The Rostock Parkinson’s disease (ROPAD) study is an observational clinical study aiming to determine the frequency and spectrum of genetic variants contributing to PD in a large international cohort.

We investigated variants in 50 genes with either an established relevance for PD or possible phenotypic overlap in a group of 12 580 PD patients from 16 countries [62.3% male; 92.0% White; 27.0% positive family history (FH+), median age at onset (AAO) 59 years] using a next-generation sequencing panel.

Altogether, in 1864 (14.8%) ROPAD participants (58.1% male; 91.0% White, 35.5% FH+, median AAO 55 years), a PD-relevant genetic test (PDGT) was positive based on *GBA1* risk variants (10.4%) or pathogenic/likely pathogenic variants in *LRRK2* (2.9%), *PRKN* (0.9%), *SNCA* (0.2%) or *PINK1* (0.1%) or a combination of two genetic findings in two genes (∼0.2%). Of note, the adjusted positive PDGT fraction, i.e. the fraction of positive PDGTs per country weighted by the fraction of the population of the world that they represent, was 14.5%. Positive PDGTs were identified in 19.9% of patients with an AAO ≤ 50 years, in 19.5% of patients with FH+ and in 26.9% with an AAO ≤ 50 years and FH+. In comparison to the idiopathic PD group (6846 patients with benign variants), the positive PDGT group had a significantly lower AAO (4 years, *P* = 9 × 10^−34^). The probability of a positive PDGT decreased by 3% with every additional AAO year (*P* = 1 × 10^−35^). Female patients were 22% more likely to have a positive PDGT (*P* = 3 × 10^−4^), and for individuals with FH+ this likelihood was 55% higher (*P =* 1 × 10^−14^). About 0.8% of the ROPAD participants had positive genetic testing findings in parkinsonism-, dystonia/dyskinesia- or dementia-related genes.

In the emerging era of gene-targeted PD clinical trials, our finding that ∼15% of patients harbour potentially actionable genetic variants offers an important prospect to affected individuals and their families and underlines the need for genetic testing in PD patients. Thus, the insights from the ROPAD study allow for data-driven, differential genetic counselling across the spectrum of different AAOs and family histories and promote a possible policy change in the application of genetic testing as a routine part of patient evaluation and care in PD.

## Teaser

Westenberger *et al.* study the frequency and spectrum of monogenic causes of Parkinson’s disease in a multiethnic sample of more than 12 000 patients, and find that about 15% harbour potentially actionable genetic variants. In the emerging era of gene-targeted trials, these findings underline the need for genetic testing in Parkinson’s disease.

**See Gan-Or *et al*. (https://doi.org/10.1093/brain/awae181) for a scientific commentary on this article.**

## Introduction

The genetic landscape of Parkinson’s disease (PD) and related phenotypes is multifaceted. Even when considering only monogenic causes of classical PD, pathogenic variants in seven genes (*LRRK2*, *PRKN*, *PINK1*, *SNCA*, *PARK7*, *VPS35* and *CHCHD2*) are implicated.^1^ Furthermore, heterozygous changes in *GBA1* are a strong risk factor for PD. In addition, >30 other more complex monogenic movement disorders may present with atypical parkinsonism or may have parkinsonism as a prominent or even predominant clinical feature in at least a subset of patients.^1^ Estimates of the spectrum of pathogenic variants in PD and their frequencies in different populations are currently limited and typically biased.^2^ Namely, PD genetic studies are either focused on targeted sequencing of a handful of the most relevant PD genes in relatively large patient groups^3-5^ or wider-range (next-generation sequencing-based) screening in small patient groups, typically of selected ethnic background.^6-9^ The hitherto largest study analysed 23 known PD genes in ∼1600 Chinese patients,^9^ whereas the first results on 1300 patients from the Rostock Parkinson’s disease (ROPAD) study were based on an international, multicentre approach.^10^ Paradoxically, a large proportion of genetic testing results in PD escape publication, because they are the product of an increasing number of commercial genetic tests performed using PD diagnostic panels or exomes. Of note, therapeutic modification of several genetic targets (most prominently *GBA1* and *LRRK2*) has reached the clinical trial stage. However, a major obstacle in conducting clinical trials is that PD patients are unaware of their genetic status and, therefore, cannot be recruited. Expanding the number of investigated PD-related genes and including additional confirmed and candidate genes related to neurological disorders with overlapping clinical features in large and well-phenotyped PD patient groups is of paramount importance for capturing the full variant spectrum underlying and/or modifying PD and for stratifying and prioritizing patients for gene-targeted clinical trials. The aim of the ROPAD study was to close current knowledge gaps by determining the frequency and spectrum of PD genetic causes across a comprehensive list of PD and other neurological disorder genes in a group of >12 500 PD patients, with a particular view towards the emerging era of gene-targeted clinical trials.

## Materials and methods

### General settings

The ROPAD study is an observational clinical study assessing the frequency and type of pathogenic variants in known PD-related genes and genes related to other movement disorders or dementia in a multicentre, international setting.^10^ The study was approved by the Ethics Committee at the Medical Faculty of the University of Rostock (A2019-0017) and by central and local institutional review boards and ethics committees of participating sites and was conducted in accordance with the Declaration of Helsinki. The ROPAD study is registered at www.clinicaltrials.gov (NCT03866603). It is part of a scientific collaboration between CENTOGENE GmbH (Rostock, Germany), the University of Lübeck (Lübeck, Germany) and Denali Therapeutics Inc. (San Francisco, CA, USA).

### Study participants

The investigated patient group consisted of 12 580 reportedly unrelated individuals (index patients) with a clinical diagnosis of PD and an age of ≥18 years (Supplementary material, Methods). Study participants were recruited at movement disorder centres in 16 different countries belonging to four wider geographical regions (Europe, the Middle East and North and South America; Supplementary Table 1) between April 2019 and May 2021. All participants underwent a neurological and movement disorder examination, medical and family history interview and collection of a dried blood spot sample.

### Genetic analyses

**Figure 1:**
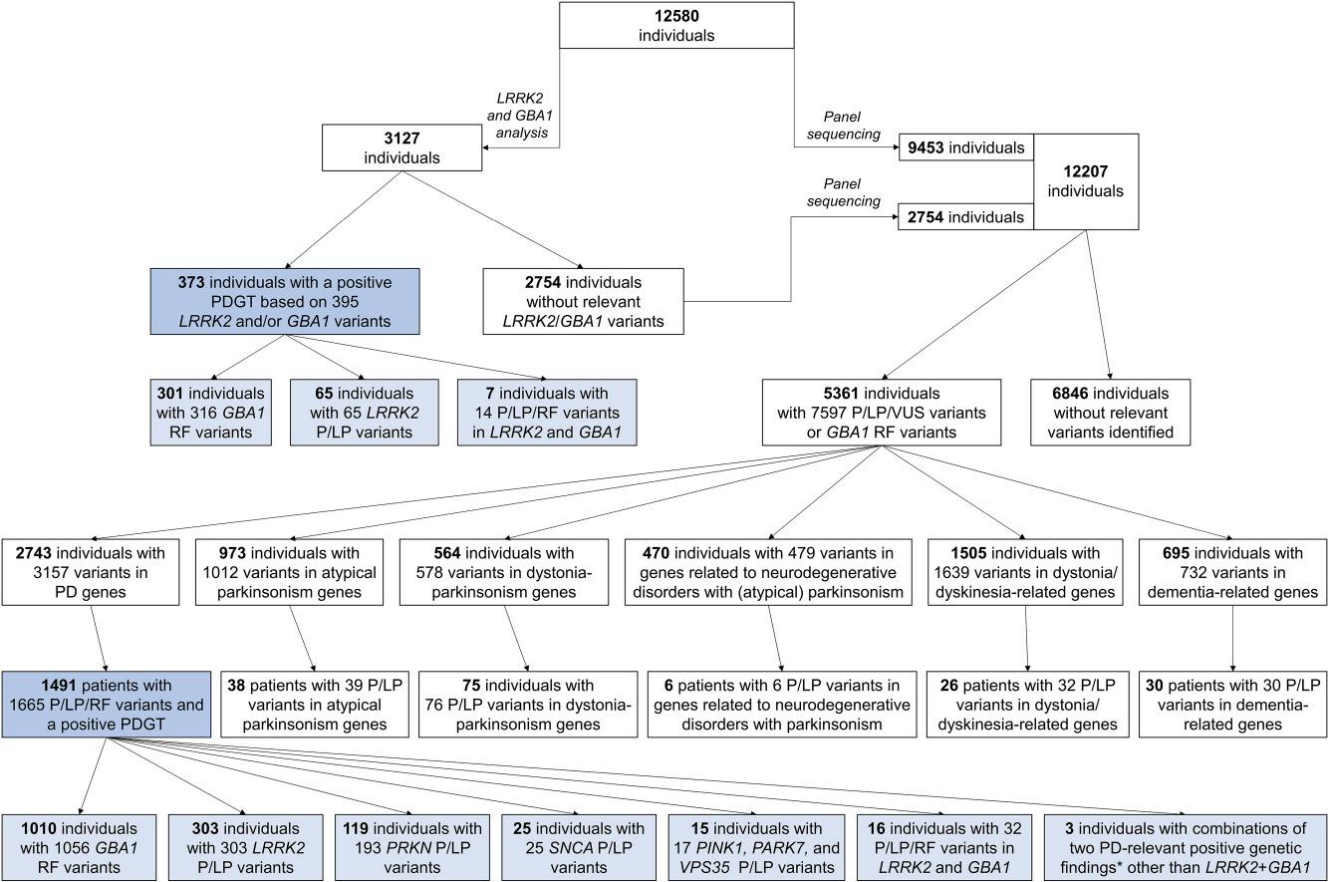
**Workflow for genetic analysis of the ROPAD study participants, indicating the numbers of analysed patients and the most relevant results.** Numbers of individuals with variants in: (i) Parkinson’s disease (PD)-related genes (*n* = 2743); (ii) atypical parkinsonism genes (*n* = 973); (iii) dystonia–parkinsonism genes (*n* = 564); (iv) genes related to neurodegenerative disorders with prominent/predominant (atypical) parkinsonism (*n* = 470); (v) dystonia/dyskinesia-related genes (*n* = 1505); and (vi) dementia-related genes (*n* = 695) do not add up to the number of individuals with pathogenic (P)/likely pathogenic (LP)/PD-relevant risk factors (RFs)/variants of uncertain significance (VUS) detected by gene panel sequencing (*n* = 5361), given that these groups of patients partly overlap (e.g. some of the patients with variants in dystonia/dyskinesia-related genes also harbour variants in PD-related genes, etc.).

Genomic DNA was extracted from dried blood spot samples and analysed at CENTOGENE GmbH. In the first subgroup of participants (*n* = 3127; Fig. 1), the presence of 11 pathogenic or likely pathogenic *LRRK2* variants and the *GBA1* coding sequence were examined, as previously reported.^10,11^ Individuals for whom this analysis did not yield a PD-relevant positive genetic finding (*n* = 2754) and the remaining 9453 ROPAD participants underwent further in-house-developed next-generation sequencing gene panel testing (Fig. 1). The panel targeted 50 genes (Supplementary Table 2), eight of which (*LRRK2*, *GBA1*, *PRKN*, *PINK1*, *PARK7*, *SNCA*, *VPS35* and *CHCHD2*) have an established relevance for PD according to the recommendations of the International Parkinson and Movement Disorder Society task force,^1^ and are hereinafter referred to as PD-related genes. The remaining genes are related to disorders that usually present with various phenotypes, including prominent/predominant parkinsonism [atypical parkinsonism, dystonia–parkinsonism, neurodegenerative disorders that may include (atypical) parkinsonism] or disorders with possible phenotypic overlap with PD/parkinsonism (dystonia/dyskinesia and dementia). All variants were classified according to the American College of Medical Genetics and Genomics criteria between July 2015 and May 2022 (Supplementary material, Methods).^12^ In genes other than *GBA1*, we considered only variants scored as pathogenic (P), likely pathogenic (LP) or variants of uncertain significance (VUS) for further analyses. In *GBA1*, we considered as PD-relevant risk factors (RFs) all variants scored as P or LP with respect to Gaucher’s disease (GD) and the two PD risk variants that do not cause GD (p.Glu365Lys and p.Thr408Met). Interpretation of the findings was performed in the clinical context. All patients with at least one P/LP variant in an autosomal dominant (AD) PD-related gene (*LRRK2*, *SNCA*, *VPS35* and *CHCHD2*) or at least two heterozygous or one homozygous P/LP variant in an autosomal recessive (AR) PD-related gene (*PARK7*, *PINK1* and *PRKN*) received a positive Clinical Laboratory Improvement Amendments-certified PD-relevant genetic test (PDGT) report. A positive PDGT report was also issued to all carriers of RFs in *GBA1*. Reports with unclear findings were provided to patients with two heterozygous variants in one AR PD-related gene where one variant was classified P/LP and the second VUS, and negative reports were supplied to patients with no relevant variant identified. Patients who consented to receive secondary findings were provided with a report with a positive genetic testing finding in other genes.

For copy-number variant (CNV) selection, we considered: (i) homozygous deletions (zero copy number); (ii) heterozygous deletions (one copy) and duplications (more than two copies), variants found with a minor allele frequency of <2% in the CENTOGENE Bio/Databank and affected more than two exons. The CNV detection algorithm has a sensitivity of >95% for all homozygous deletions and heterozygous deletions/duplications spanning at least three consecutive exons, based on the internal validation data set of 150 individuals. In the present cohort, CNVs were evaluated by visual inspection of the raw data in the integrative genome viewer. Typically, homozygous variants did not require confirmation by an orthogonal method. Heterozygous CNVs were confirmed by qPCR or multiplex ligation-dependent probe amplification.

For a subgroup of 2587 patients with no reportable genetic finding after PD panel sequencing, whole genome sequencing was carried out: (i) to confirm absence of relevant diagnostic findings; (ii) to define cohort ethnicity based on characteristic genotypes; and (iii) to search for new genetic causes or modifiers of PD (Supplementary material, Methods).

### Statistical analyses

All statistical analyses were performed in R (v.4.1.2). Continuous variables are visualized using box plots, whereas categorical variables are shown in stacked bar plots. Pairwise comparisons were calculated using the non-parametric two-sample Wilcoxon test for continuous variables, because all continuous variables showed deviations from a normal distribution (Supplementary Fig. 1). For categorical variables, Pearson’s χ^2^ test was calculated. Overall, five different variables [age at onset (AAO), age at clinical diagnosis (AAD), age at enrolment (AAE), sex and positive family history] were investigated for group differences in genetic subgroups, leading to a total of 130 statistical tests being performed. The global significance threshold was set to 0.05/130 = 0.0003846 based on Bonferroni correction. Therefore, any *P*-value of <3.85 × 10^−4^ was assumed to be statistically significant. To adjust additionally for the effects of sex and family history on AAO, AAD and AAE, we also calculated adjusted *P*-values for these comparisons by estimating linear models adjusted for these two categorical variables. Given that we consider these only as secondary analyses, we did not include these additional 130 *P*-values in our Bonferroni correction. To examine the association between AAO and the probability of a positive PDGT, a logistic regression model adjusted for sex and family history was calculated, and probabilities for all possible AAOs of 1–100 years were estimated.

## Results

### Study participants

**Figure 2:**
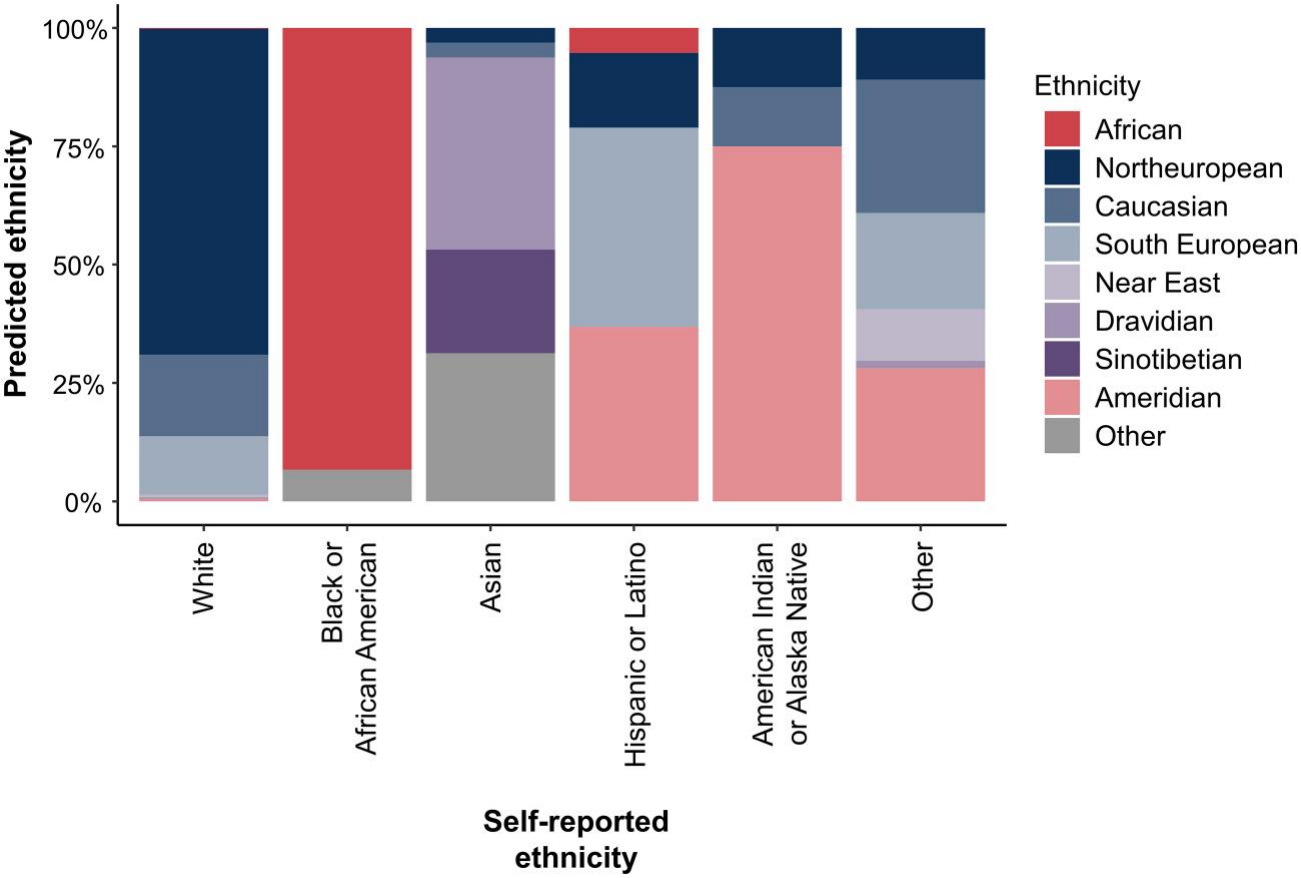
Ethnicity predicted based on the available whole genome sequencing data versus self-reported ethnicity from a subgroup of 2587 ROPAD participants.

The majority of the 12 580 ROPAD patients (62.3% male) were European (46%) and White (92%) (Table 1, Supplementary Table 3 and Supplementary Figs 2 and 3). Of note, the self-reported ethnicity and the ethnicity predicted based on the available whole genome sequencing data in a subgroup of ROPAD participants matched closely (Fig. 2). The median AAO, AAD and AAE of study participants were 59 years [interquartile range (IQR), 50–66 years; range, 1–94 years], 60 years (IQR, 52–68 years; range, 1–94 years) and 67 years (IQR, 59–74 years; range, 18–95 years), respectively (Table 1). Twenty-seven per cent of individuals reported positive family history.

**Table 1:** Demographic and clinical data of the ROPAD patient cohort and different genetic subgroups

Characteristic		All study participants	PDGT-positive group^a^	Idiopathic group^b^	*GBA1*-related PDGT-positive group	*LRRK2*-related PDGT-positive group	*LRRK2 + GBA1-*related PDGT-positive group	*PRKN*-, *PINK1*- or *PARK7*-related PDGT-positive group	*SNCA*-related PDGT-positive group
Number of patients		12 580	1864	6846	1311	368	23	132	25
Sex	Male (%)	7840 (62.3)	1083 (58.1)	4315 (63.0)	793 (60.5)	189 (51.4)	9 (39.1)	75 (56.8)	16 (64.0)
	Female (%)	4740 (37.7)	781 (41.9)	2531 (37.0)	518 (39.5)	179 (48.6)	14 (60.9)	57 (43.2)	9 (36.0)
Ethnicity	White	11 579 (92.0)	1697 (91.0)	6369 (93.0)	1212 (92.4)	319 (86.7)	19 (82.6)	118 (89.4)	24 (96.0)
	Asian	139 (1.1)	20 (1.1)	63 (0.9)	9 (0.7)	8 (2.2)	0 (0.0)	3 (2.3)	0 (0.0)
	Hispanic or Latino	49 (0.4)	4 (0.2)	34 (0.5)	2 (0.2)	1 (0.3)	0 (0.0)	1 (0.8)	0 (0.0)
	Black or African American	149 (1.2)	16 (0.9)	79 (1.2)	14 (1.1)	1 (0.3)	0 (0.0)	1 (0.8)	0 (0.0)
	Native Hawaiian or Other Pacific Islander	5 (0.0)	0 (0.0)	4 (0.1)	0 (0.0)	0 (0.0)	0 (0.0)	0 (0.0)	0 (0.0)
	American Indian or Alaska Native	32 (0.3)	3 (0.2)	13 (0.2)	2 (0.2)	1 (0.3)	0 (0.0)	0 (0.0)	0 (0.0)
	Other	619 (4.9)	122 (6.5)	281 (4.1)	71 (5.4)	37 (10.1)	4 (17.4)	9 (6.8)	1 (4.0)
	Unknown	8 (0.1)	2 (0.1)	3 (0.0)	1 (0.1)	1 (0.3)	0 (0.0)	0 (0.0)	0 (0.0)
Median age	At onset	59 years (IQR: 50–66 years; range: 1–94 years; *n* = 12 575)	55 years (IQR: 46–63 years; range: 2–89 years; *n* = 1863)	59 years (IQR: 50–67 years; range: 1–94 years; *n* = 6844)	56 years (IQR: 48–63 years; range: 2–89 years; *n* = 1310)	58 years (IQR: 50–65 years; range: 6–89; *n* = 368)	56 years (IQR: 48–63 years; range: 34–83; *n* = 23)	35 years (IQR: 28–43 years; range: 9–74; *n* = 132)	50 years (IQR: 38–54 years; range: 18–66; *n* = 25)
	At clinical diagnosis	60 years (IQR: 52–68 years; range: 1–94 years; *n* = 12 573)	57 years (IQR: 48–64 years; range: 3–90 years; *n* = 1863)	61 years (IQR: 53–68 years; range: 1–94 years; *n* = 6842)	58 years (IQR: 49–65 years; range: 3–90 years; *n* = 1310)	59 years (IQR: 52–67 years; range: 30–90; *n* = 368)	59 years (IQR: 50–64 years; range: 34–83; *n* = 23)	38 years (IQR: 31–46 years; range: 11–74; *n* = 132)	50 years (IQR: 41–55 years; range: 19–69; *n* = 25)
	At enrolment	67 years (IQR: 59–74 years; range: 18–95; *n* = 12 579)	64 years (IQR: 56–72 years; range: 18–91; *n* = 1864)	68 years (IQR: 60–74 years; range: 19–95; *n* = 6846)	64 years (IQR: 57–71 years; range: 21–90; *n* = 1311)	68 years (IQR: 60–74 years; range: 30–91; *n* = 368)	67 years (IQR: 61–75 years; range: 40–89; *n* = 23)	54 years (IQR: 44–61 years; range: 18–77; *n* = 132)	55 years (IQR: 47–65 years; range: 19–73; *n* = 25)
Family history	Positive	3394 (27.0)	661 (35.5)	1744 (25.5)	406 (31.0)	161 (43.8)	10 (43.5)	63 (47.7)	16 (64.0)
	Negative	8779 (69.8)	1139 (61.1)	4880 (71.3)	855 (65.2)	194 (52.7)	13 (56.5)	68 (51.5)	9 (36.0)
	Unknown	407 (3.2)	64 (3.4)	222 (3.2)	50 (3.8)	13 (3.5)	0 (0.0)	1 (0.8)	0 (0.0)

IQR = interquartile range; PD = Parkinson's disease; PDGT = Parkinson's disease-relevant genetic test.

^a^Patients with a positive PDGT.

^b^Patients without pathogenic variants/likely pathogenic variants/variants of uncertain significance identified by gene panel sequencing.

### Genetic analyses

#### Targeted *GBA1* and *LRRK2* analysis and panel sequencing findings in PD-related genes

Our initial strategy was to analyse the most likely candidates, i.e. genetic variants in *LRRK2* and *GBA1*. Hereby, 3127 individuals were investigated, yielding positive PDGTs in 373 (11.9%) participants (Fig. 1, Supplementary Tables 4 and 5 and Supplementary material ‘Results’).

Apart from 301 (9.6%) and 65 (2.1%) patients who had a *GBA1*- and *LRRK2*-related positive PDGT, respectively, seven individuals (0.2%) harboured a combination of one *GBA1* and one *LRRK2* variant (denoted as *LRRK2* + *GBA1*).

Among the 12 207 ROPAD participants investigated by panel sequencing, we detected a total of 3157 variants (627 unique variants) in the eight PD-related genes in 2743 (22.5%) patients (Fig. 1). Of those variants, 686 (64 unique) were scored as P, 191 (55 unique) as LP, 1205 (434 unique) as VUS, and 1075 (75 unique) as RF in *GBA1* (Supplementary Table 6). As a result, 1491 patients (12.2%) harbouring a total of 1813 variants (260 unique), of which 1665 were P/LP/RF (167 unique) in PD-related genes, received reports with their positive PDGT finding (Fig. 1, Supplementary Fig. 4, Supplementary material, Results and Supplementary Tables 6 and 7). Separated per gene, 1010 (8.3%), 303 (2.5%), 119 (1.0%) and 25 (0.2%) individuals screened by gene panel had a positive PDGT with a finding in *GBA1*, *LRRK2*, *PRKN* and *SNCA*, respectively. Variants in *PINK1*, *PARK7* and *VPS35* resulted in positive PDGTs in a total of 15 (0.1%) individuals. Of note, 19 (0.2%) study participants had P/LP/RF variants in two genes in various combinations, either one of which would have resulted in a positive PDGT report (Fig. 1 and Supplementary Table 7).

Altogether, we detected 297 (66 unique) CNVs in PD-related genes (Supplementary Table 6). The highest total number of CNVs (200; 42 unique) was found in *PRKN*, and there were 17 (whole gene amplification) CNVs in *SNCA*, eight (five unique) CNVs in *PARK7* and four (three unique) CNVs in *PINK1* (Supplementary Table 6). In *GBA1*, we discovered 66 (13 unique) recombinations. The two (one unique) CNVs in *VPS35* were not considered pathogenic (Supplementary Table 6). Among the P/LP/RF variants in 1491 patients who had a panel sequencing-based positive PDGT, 194 (51 unique; 11.7%) were CNVs, whereas 1471 (116 unique; 88.3%) were short sequence variants (SSVs; single-nucleotide variants and indels) (Supplementary Table 8).

Findings of the 1252 (10.3%) patients who carried potentially relevant variants in PD-related genes, including VUS or heterozygous variants in AR PD genes, that did not suffice to render a positive PDGT report are shown in Supplementary Table 9.

Overall, 1864 (14.8%) ROPAD participants had a positive PDGT (Table 2). *LRRK2*, GD-related *GBA1* and *PRKN* variants accounted for a significantly higher (*P* < 0.00001) percentage of familial than sporadic PD patients (*LRRK*2, 5.04% versus 2.36%; GD-related *GBA1* variants, 7.04% versus 4.84%; *PRKN*, 1.68% versus 0.72%, respectively; Supplementary Table 10). The two PD-related *GBA1* RFs were found in comparable percentages of patients with a positive and negative family history (p.Glu365Lys, 2.95% versus 3.09%; p.Thr408Met, 2.92% versus 2.31%, respectively; Supplementary Table 10).

**Table 2:** ROPAD study participants who received a positive Parkinson’s disease-relevant genetic test: overview per gene

Gene	Number of patients with a positive PDGT related to respective genes	Percentage (%)
*GBA1*	1311	10.42
*LRRK2*	368	2.93
*PRKN*	119	0.95
*SNCA*	25	0.20
*PINK1*	9	0.07
*PARK7*	4	0.03
*CHCHD2*	0	0.00
*VPS35*	2	0.02
*GBA1 + LRRK2*	23	0.18
*GBA1 + PRKN*	2	0.02
*GBA1 + VPS35*	1	0.01
Sum:	1864	14.82

PDGT = Parkinson’s disease-relevant genetic test.

#### Findings in other investigated genes

The results of panel sequencing of the 42 genes not related to classical PD are shown in Table 3 and Supplementary Tables 6 and 7. Briefly, we identified 4440 P/LP/VUS variants (2163 unique; Supplementary Table 6) in 3573 (30.3%) individuals. Of these, 530 (14.8%) patients had an additional positive PDGT and 78 (2.2%) had at least one P/LP variant in some of the AR PD genes. Altogether, 98 individuals (0.8% of those tested by gene panel analysis) received a positive genetic testing finding for the respective gene (Table 3), including 12 who also had positive PDGTs (Supplementary Table 7). Of note, *GCH1* was the gene with most of the P/LP variants among the other investigated genes, i.e. heterozygous *GCH1* variants were found in 30 patients, 6 of whom also had findings in other genes (PD-related and *PDE8B*, respectively; Table 3 and Supplementary Table 7).

**Table 3:** Overview of variants in the ROPAD study participants who received positive genetic testing reports owing to findings in genes not related to classical Parkinson’s disease

Gene	Number of patients who received a report with a PGT finding in respective genes	Percentage of patients tested by gene panel analysis (%)
**Atypical parkinsonism**		
*ATP13A2*	1	0.01
*RAB39B*	1	0.01
*SYNJ1*	1	0.01
**Dystonia–parkinsonism**		
*GCH1*	24	0.20
*C19orf12*	1	0.01
*PLA2G6*	3	0.02
**Neurodegenerative disorders that may include (atypical) parkinsonism**		
*PDE8B*	1	0.01
*PDGFB*	1	0.01
*PDGFRB*	2	0.02
*SLC20A2*	1	0.01
**Dystonia–parkinsonism + a neurodegenerative disorder that may include (atypical) parkinsonism**		
*GCH1 + PDE8B*	1	0.01
**Dystonia/dyskinesia**		
*GNAL*	3	0.02
*KMT2B*	1	0.01
*SGCE*	8	0.07
*THAP1*	1	0.01
*TOR1A*	9	0.07
**Dementia**		
*APP*	1	0.01
*GRN*	18	0.15
*MAPT*	4	0.03
*PSEN1*	2	0.02
*PSEN2*	2	0.02
*Sum*	86	0.73

PGT = positive genetic testing.

### Comparison between monogenic and idiopathic PD patients and between different genetic PD subtypes

#### Idiopathic PD and PDGT-positive group

The 6846 patients in whom only benign and likely benign variants were identified represent the most likely non-monogenic (‘idiopathic’) PD patient group (IPD). Therefore, we compared sex, family history, AAO, AAD and AAE between this group of patients and individuals with a positive PDGT (‘PDGT-positive group’; *n* = 1864) (Table 1).

**Figure 3:**
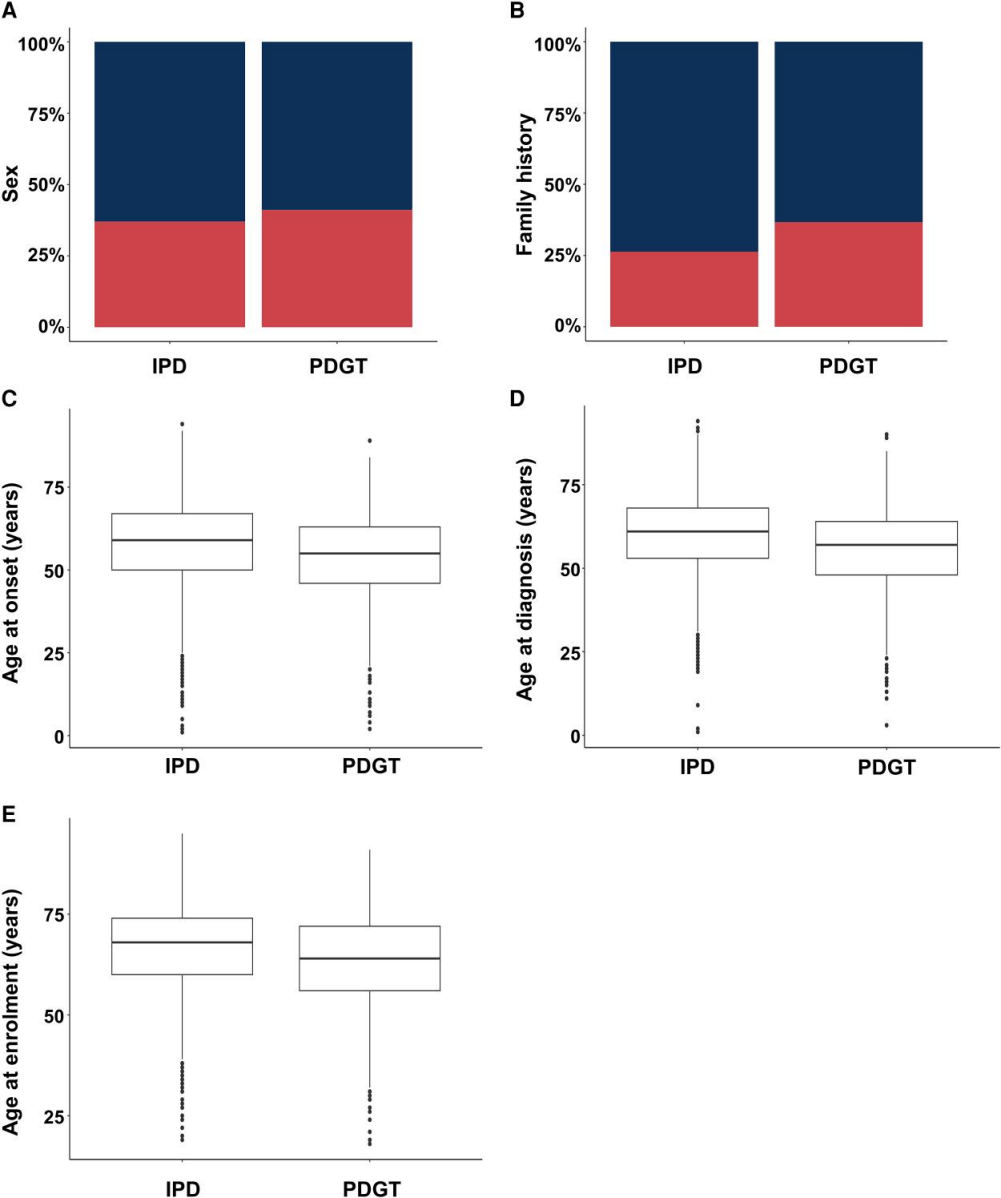
**Comparison of demographic and age-related variables between idiopathic Parkinson’s disease patients and those with a positive Parkinson’s disease-relevant genetic test (PDGT)**. (**A**) Sex (*top section* in blue = male; *bottom section* in red = female). (**B**) Fractions of patients with positive family history (*bottom section* in red = positive family history; *top section* in blue = negative family history). (**C**) Age at onset. (**D**) Age at diagnosis. (**E**) Age at enrolment. IPD = idiopathic Parkinson’s disease.

Sex and family history were significantly associated (*P* = 1.12 × 10^−4^ and *P* = 5.90 × 10^−18^, respectively) with the PDGT-positive group (Table 1, Fig. 3A and B and Supplementary Table 11). Specifically, the male-to-female ratio in the PDGT-positive group was lower than in the IPD group (1.4 versus 1.7), and female patients were 22% more likely to have a positive PDGT [*P* = 3.02 × 10^−4^, odds ratio (OR): 1.22]. The fraction of individuals with a positive family history was higher in the PDGT-positive group compared with the IPD group (35.5% versus 25.5%), and patients with a positive family history were 55% more likely to have a positive PDGT (*P* = 1.41 × 10^−14^, OR: 1.55) than those with a negative family history.

The median AAO, AAD and AAE of the PDGT-positive group were each 4 years lower than in the IPD group (*P* = 8.94 × 10^−34^, *P* = 3.28 × 10^−36^ and *P* = 2.45 × 10^−33^, respectively; Table 1, Fig. 3C–E and Supplementary Table 11). Stratifying the analyses by the geographical regions from which the patients were recruited showed the same trends (Supplementary Fig. 5). The probability of having a positive PDGT decreased with advancing AAO (3% for every additional year of AAO, *P* = 1.04 × 10^−35^, OR: 0.97; Table 4, Supplementary Fig. 6 and Supplementary Table 12). In the subgroup of all ROPAD patients with AAO ≤ 50 years (*n* = 3445), 19.9% (*n* = 684) had a positive PDGT report (Table 4). In the subgroup of patients with a positive family history (*n* = 3394), 661 (19.5%) had a positive PDGT. Among patients who had AAO ≤ 50 years and positive family history (*n* = 1033), 26.9% (*n* = 278) received a positive PDGT report.

**Table 4:** Distribution of family history findings and ages at onset among the ROPAD study participants and patients with a positive Parkinson’s disease-relevant genetic test

	All ROPAD study participants	Patients with a positive PDGT		ROPAD study participants with positive family history	Patients with positive family history and a positive PDGT			
AAO (years)	*n* (%)	*n* (%)	ROPAD participants of the respective AAO range (%)	*n* (%)	*n* (%)	Percentage of the ROPAD study participants	Percentage of the ROPAD study participants with positive family history	Percentage of all individuals with a positive PDGT
≤20	73 (0.6)	27 (1.4)	37.0	31 (0.9)	15 (2.3)	20.5	48.4	0.8
≤30	261 (2.1)	79 (4.2)	30.3	100 (2.9)	39 (5.9)	14.9	39.0	2.1
≤40	1145 (9.1)	269 (14.4)	23.5	367 (10.8)	119 (18.0)	10.4	32.4	6.4
≤50	3445 (27.4)	684 (36.7)	19.9	1033 (30.4)	278 (42.1)	8.1	26.9	14.9
All	12 580 (100)	1864 (100)	14.8	3394 (100)	661 (100)	5.3	19.5	35.5

AAO = age at onset; PDGT = Parkinson’s disease-relevant genetic test.

#### Idiopathic PD and genetic subgroups

**Figure 4:**
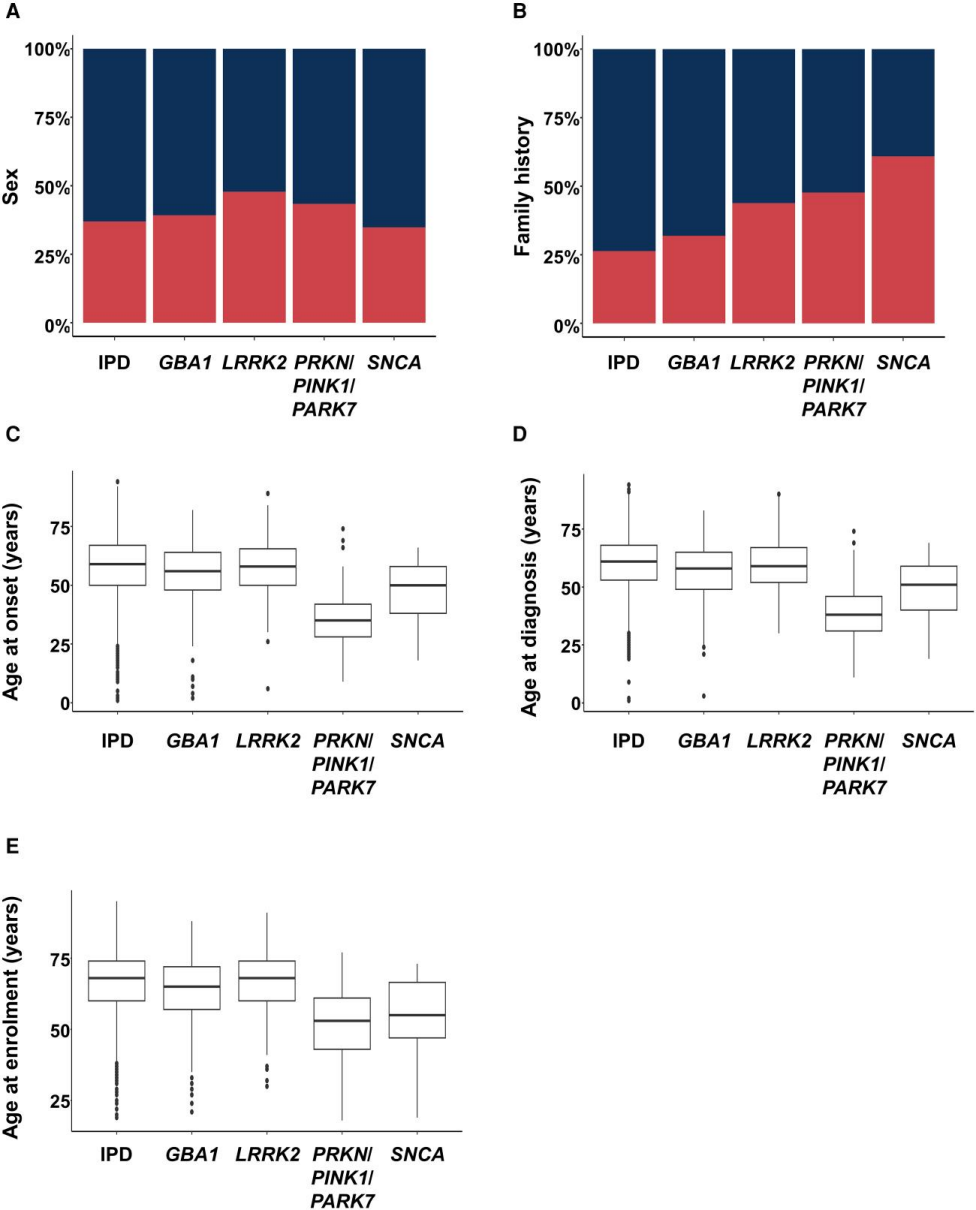
**Comparison of demographic and age-related variables between idiopathic Parkinson’s disease patients and four different genetic patient subgroups [positive Parkinson’s disease-relevant genetic test (PDGT) based on *GBA1*, *LRRK2*, *PRKN*/*PINK1* or *SNCA* variants].** (**A**) Sex (*top section* in blue = male; *bottom section* red = female). (**B**) Fractions of patients with positive family history (*bottom section* in red = positive family history; *top section* in blue = negative family history). (**C**) Age at onset. (**D**) Age at diagnosis. (**E**) Age at enrolment. IPD = idiopathic Parkinson’s disease.

Demographic and age-related features comparing IPD patients and four gene-stratified patient subgroups (with positive PDGTs related to *GBA1*, *LRRK2*, *PRKN*/*PINK1*/*PARK7* or *SNCA*) are given in Fig. 4, Supplementary Fig. 7 and Supplementary Tables 1 and 11. A significant difference with respect to sex was observed only between the *LRRK2*-related PDGT-positive and IPD subgroups. The fraction of individuals with a positive family history was significantly higher in the *LRRK2-*, *PRKN*/*PINK1*/*PARK7*- and *SNCA*-related PDGT-positive subgroups compared with the IPD group (Supplementary Table 11). AAO, AAD and AAE were significantly lower in patients with a *PRKN*/*PINK1*/*PARK7*-related positive PDGT in comparison to the IPD and all other genetic groups apart from the *SNCA*-related PDGT-positive group. Also, AAO, AAD and AAE were significantly lower in the *GBA1-*related PDGT-positive subgroup compared with the IPD patients. There was a lower proportion of patients with a positive family history and lower AAE in patients with *GBA1* variants than in those with *LRRK2* variants.

#### Idiopathic PD and *LRRK2*, *GBA1* and *LRRK2* + *GBA1* subtypes

We next explored potential differences between patients with a positive PDGT based on findings in *GBA1* (p.Glu365Lys, p.Thr408Met or GD-relevant *GBA1*), *LRRK2* or both (*LRRK1* + *GBA1*) (Supplementary Figs 8, 9 and Supplementary Tables 1 and 11). This analysis excluded patients with additional P/LP variants in other PD-, parkinsonism- or dementia-related genes. Pairwise comparison between IPD patients and individuals with a positive PDGT based on the p.Glu365Lys, p.Thr408Met or GD-relevant *GBA1* variants revealed no significant differences in AAO between IPD and carriers of p.Glu365Lys or p.Thr408Met. The p.Glu365Lys group (and not p.Thr408Met) had a significantly higher AAD and AAE than patients with the GD-relevant *GBA1* variants. All three age categories were significantly lower, and family history was more likely to be positive in patients with GD-relevant *GBA1* variants compared with the IPD group. Furthermore, we observed a significantly lower AAO, AAD and AAE in patients with GD-relevant *GBA1* variants in comparison to the *LRRK2*-related PDGT-positive group.

## Discussion

The aetiology of PD is multifactorial, even in seemingly monogenic forms. This is evident from the considerably reduced penetrance of *LRRK2* pathogenic variants that might be explained, in part, by environmental factors^13^ or genetic modifiers.^14,15^ Age-related reduced penetrance is also observed in carriers of *GBA1* variants, although *GBA1* variants are generally considered the most important genetic risk factor for PD rather than a monogenic cause.^16,17^ The consideration of the level to which a monogenic (mono- or biallelic variant in AD or AR genes, respectively) variant contributes to the disease occurrence questions the very concept of ‘monogenic PD’ and, consequently, terms such as ‘molecular diagnosis’. To circumvent this issue and report our findings as objectively as possible and without defining them as decidedly ‘disease-causing’, we coined the term ‘positive PDGT’.

The ROPAD study pilot findings from 1288 initially recruited patients resulted in ∼13% study participants with a positive PDGT attributable to *GBA1* (∼9%), *LRRK2* (∼3%) and *PRKN* (∼1%) variants.^10^ Analysis of the complete ROPAD data set revealed an even greater positive PDGT yield, indicating that in ∼15% of patients, PD has a genetic contribution that is mostly conferred by variants in *GBA1* (∼10%), *LRRK2* (∼3%) or *PRKN* (∼1%). When considering the family history of patients with a positive PDGT report, >5% of familial and >2% of sporadic ROPAD patients harboured P/LP variants in *LRRK2*. Variants in *PRKN* accounted for 1.7% of familial and 0.7% of sporadic patients. The slightly higher percentage of patients with *GBA1* variants seen among patients with a positive family history (>12%) versus those with a negative family history (10%) is largely driven by the GD-related *GBA1* variants (Supplementary Table 10).

Of note, more than two-thirds of the ROPAD patients were recruited at tertiary referral centres, which might have led to a slight overestimation of the genetic contribution, although the mean AAO or percentage of participants with a positive family history did not seem to differ considerably from those in even more unselected samples. Among the eight countries each contributing >500 patients, Israel and Spain had the highest percentage of individuals with a positive PDGT report out of initially recruited patients (19.5% and 18.2%, respectively). These numbers are likely to be influenced by the Ashkenazi Jewish and Berber populations, in which the frequency of *GBA1* and *LRRK2* variants are increased in comparison to other populations, and might thus inflate the level of genetic contribution in PD in our study. Therefore, we weighted the fraction of positive PDGTs per country by the fraction of the population of the world that they represent (e.g. patients from Israel represent 10% of ROPAD participants, whereas Israelis constitute 0.1% of the population of the world; Supplementary Fig. 10). This resulted in an adjusted fraction of 14.5% (versus 14.8% of positive PDGT findings in ROPAD), indicating that no substantial inflation of positive results occurs in our study. In contrast, a fraction of individuals who harboured one or a combination of P, LP or VUS variants in PD-related genes not currently fulfilling positive PDGT criteria might, conceivably, still receive such a report in the future, because some of the variants might be reclassified from VUS to LP/P variants.^18^ This was the case even during the course of the ROPAD study (Supplementary material, Results), underlining the necessity of revisiting genetic testing results regularly and highlighting the enormous potential of individualized patient care. Furthermore, our next-generation sequencing panel designed 5 years ago consisted only of genes deemed relevant for PD and parkinsonism at that time. As more genes are continually being linked to monogenic parkinsonism, our results conceivably represent an underestimation of the frequency of genetic PD forms. Thus, neither variant nor disease gene classification is fixed over time, and they are subject to continuous re-evaluation and, if needed, reclassification. In a follow-up study, patients with negative genetic findings who consented to further analyses will undergo whole genome sequencing in search of novel PD genetic causes/modifiers. Of note, among the first 2587 patients analysed in this way, we identified only five patients harbouring single-exon *PRKN* CNVs *in trans* not previously detected by panel sequencing. Given that this was explained fully by technical parameters defined in our panel-analysis pipeline, a very small fraction of patients is underdiagnosed based on current diagnostic evidence. When extrapolating these findings to the entire ROPAD patient group with negative genetic findings upon panel sequencing, we estimate to have missed 21 positive PDGT results by our panel analysis, resulting in a sensitivity of 98.6%, in comparison to the gold-standard whole genome sequencing. Another limitation of our study is a relatively ethnically uniform PD patient population, consisting of ∼90% White individuals. Thus, to gain an even more precise insight into the global frequencies of monogenic PD causes, the collection of more admixed or currently under-represented isolated populations should be aimed for. Along the same lines, given that the ROPAD patient group includes only 139 Asian patients, it is not surprising that no *CHCHD2* variants were identified in our study. Pathogenic variants in *CHCHD2* have primarily been reported in Chinese/Asian PD patients, implying that they might be rare in other populations. Nevertheless, we retained this gene in the list of PD-related genes to comply with the recommendations of the International Parkinson and Movement Disorder Society Task Force on PD gene curation.

As expected, a positive family history was significantly more frequent in patients with (35%) in comparison to those without (25%) a positive PDGT, and individuals with a positive family history were 55% more likely to have a positive PDGT report (Table 4 and Supplementary Table 12). Apart from family history, an early AAO is the strongest indicator of a monogenic PD cause in a patient. Hence, it is not surprising that the median AAO was 4 years lower in patients with a positive PDGT in comparison to the IPD group in the ROPAD study and that the probability of having a positive PDGT decreased by 3% with every additional AAO year (Supplementary Fig. 6 and Supplementary Table 12). Positive PDGT findings were identified in 20% of patients with AAO ≤ 50 years or with a positive family history. Of note, among the ROPAD participants with a positive family history and AAO ≤ 50 years, 27% had a positive PDGT, whereas this percentage was nearly 50% among those with a positive family history and AAO ≤ 20 years (Table 4). Female patients were 22% more likely to have a positive PDGT. This is probably attributable to the inherent differences in IPD frequencies between men and women and indicates that genetic factors, inherited in Mendelian fashion, play a similar role in both sexes. Given that IPD affects more men than women, in any given patient group that consists of genetic and idiopathic PD patients, the percentage of women affected by genetic PD versus IPD will be higher than the percentage of men owing to the higher denominator (number of IPD individuals) in men.

Although association studies^19,20^ and metabolic^21,22^ and neuroimaging^21^ evidence established a relationship between the p.Glu365Lys or p.Thr408Met *GBA1* variants and PD, their contribution to PD aetiology is still vividly debated. The significantly lower AAO, AAD and AAE and the higher proportion of patients with a positive family history seen in ROPAD patients who received their *GBA1*-related positive PDGT were driven by GD-relevant *GBA1* variants and not by the p.Glu365Lys and p.Thr408Met RFs only associated with PD. This finding underlines the distinction between these two variants and GD-relevant *GBA1* variants with respect to PD aetiology. Nevertheless, both p.Glu365Lys and p.Thr408Met were detected at significantly higher frequencies in ROPAD patients in comparison to the highest population frequency in the Genome Aggregation Database, confirming their association with PD (Supplementary material, Discussion).

In contrast to the anticipated additive damaging outcome of two variants in *LRRK2* and *GBA1*, an interaction of *LRRK2* and *GBA1* variants might have a protective effect.^23-25^ Interestingly, ROPAD participants with *GBA1* or *LRRK2* + *GBA1* variants (*n* = 1311 and *n* = 23, respectively) had the same median AAO, which was 2 years earlier than in patients with only *LRRK2* variants (*n* = 368).

Thirty-seven of our patients had P/LP variants in genes related to parkinsonism that are likely to be responsible for PD symptoms in these individuals. This finding is not surprising, given the numerous conditions that mimic PD and the reports that a considerable fraction of patients clinically diagnosed with PD is not confirmed to have PD at autopsy.^26,27^ Importantly, it underlines the necessity of analysing a wide spectrum of parkinsonism-related genes in PD patients. Of note, 24 of these patients had P/LP variants in *GCH1*, a gene classified as a dystonia–parkinsonism gene based on the recommendations of the International Parkinson and Movement Disorder Society Task Force.^1^ Although *GCH1* variants have been described to date in numerous classical PD patients, making *GCH1* an excellent PD-related gene candidate, our internal gene–disease validity assessment under the ClinGen framework^28^ showed only a ‘moderate relationship’ between *GCH1* and AD PD. Nevertheless, if *GCH1* were to become an established PD-related gene in the future, this would increase the percentage of positive PDGT findings in ROPAD from 14.8% to 15.0%.

Fifty-six patients had positive genetic testing findings in genes related to dystonia/dyskinesia or dementia. Variants in none of the five implicated dystonia genes (*TOR1A*, *SGCE*, *GNAL*, *THAP1* and *KMT2B*) have been reported to cause PD/parkinsonism to date. However, an underlying association between dystonia and PD cannot be excluded entirely, given that dystonia can be part of the clinical PD spectrum and vice versa, and variants in dystonia-related genes have reduced penetrance. Considering the neurodegenerative nature of dementia and PD, their common phenotypic overlap is, likewise, not surprising, and the presence of dementia-associated variants in the PD cohort might signify a causal role of these variants in PD pathogenesis. The phenotypic spectrum of variants in *GRN* and *MAPT* often includes atypical parkinsonism that might even predominate over the clinical presentation.^1^ Interestingly, both *GRN* and *MAPT* represent PD susceptibility/risk loci discovered through association studies, and their protein products are functionally related to PD proteins.^29,30^ Thus, at least *GRN* and *MAPT* should be tested routinely in PD patients.

In general, a data-driven estimate of pathogenic variant frequency in a very large cohort unselected for family history and age at onset is the basis for the design of genetic screening studies to identify candidates for clinical trials/build up clinical trial-ready cohorts. Another advantage of high translational value is the systematic feedback of the genetic result to the patient and the offer of genetic testing in the event of a positive test. In the counselling situation, patients can then be advised of the possibility of participating in gene-specific trials. Furthermore, this approach allows the identification of other mutation carriers in affected families who could also benefit from gene-specific therapies in the future. In this context, several clinical trials are currently recruiting patients with *GBA1*-related PD (NCT02914366, NCT05830396, NCT05819359 and NCT04127578). To take trial readiness into account, the ROPAD protocol was adapted during the course of the study with an adjustment of the maximum disease duration (reduction to 5 years), which was the allowed maximum disease duration of a phase 3 trial using the *LRRK2* kinase inhibitor BIIB122 (DNL151) in patients carrying a kinase activity-increasing *LRRK2* pathogenic variant (NCT05418673). This programme was, however, replaced in the autumn of 2023 with a phase 3 trial with the same compound in patients with PD regardless of the genotype (NCT04056689). The number of trials targeting *LRRK2* and *GBA1* will increase in the future and will also be extended to genes with lower pathogenic variant frequencies. Thus, a considerable fraction of these 1864 patients carrying a pathogenic variant would be potential candidates for inclusion in ongoing or imminent clinical trials. Although not a gene-specific therapy in the narrow sense, the response to deep brain stimulation might vary depending on the underlying genotype.^31^ In addition, the postoperative risk of developing dementia appears to be higher in *GBA1*-PD than in other genetic forms, which could be included in future treatment guidelines to identify suitable candidates for deep brain stimulation and to predict the postoperative outcome better.^32^

## Conclusion

In conclusion: (i) variants in PD-related genes contribute to the disease in 15% of all PD patients; (ii) ∼90% of patients with a positive PDGT had variants in *LRRK2* or *GBA1*, making these individuals potential candidates to be included in gene-targeted trials; (iii) positive PDGT findings were identified in 20% of patients with AAO ≤ 50 years or with a positive family history, and in 27% of individuals with AAO ≤ 50 years and a positive family history, suggesting that genetic testing might be offered preferentially to these patient groups; (iv) a small but considerable number of PD patients carry pathogenic variants in genes related to dystonia/dyskinesia or dementia, raising the possibility of shared underlying pathogenetic mechanisms; and (v) the ROPAD study results inform differential genetic counselling and patient prioritization for clinical trials.

## Supplementary Material

awae188_Supplementary_Data

## Data Availability

The data that support the findings of this study are available from the corresponding author, upon reasonable request.
