# Supplementary material for: Relevance of genetic testing in the gene-targeted trial era: the Rostock Parkinson’s disease study
Source: Brain. 2024 Aug 1;147(8):2652–67. doi: 10.1093/brain/awae188 (PMC11292909; doi:10.1093/brain/awae188)
Supplement: awae188_Supplementary_Data [file awae188_supplementary_data.zip › brain-2023-02608-File010.pdf]

Supplementary Material
Relevance of genetic testing in the gene-targeted trial era: The Rostock Parkinson's Disease Study

Table of Contents

Supplementary Methods	1
Supplementary Results	3
Supplementary Discussion	5
Supplementary References	6
Supplementary Table 1	7
Supplementary Table 2	10
Supplementary Table 3	11
Supplementary Table 4	19
Supplementary Table 5	21
Supplementary Table 6	Excel file
Supplementary Table 7	22
Supplementary Table 8	23
Supplementary Table 9	24
Supplementary Table 10	25
Supplementary Table 11	26
Supplementary Table 12	30
Supplementary Figure 1	31
Supplementary Figure 2	32
Supplementary Figure 3	33
Supplementary Figure 4	34
Supplementary Figure 5	35
Supplementary Figure 6	36
Supplementary Figure 7	37
Supplementary Figure 8	38
Supplementary Figure 9	39
Supplementary Figure 10	40

Supplementary Methods

Study participants

The Rostock Parkinson's Disease (ROPAD) study group analysed in this study consisted of 12580 reportedly unrelated Parkinson's disease (PD) patients recruited at 116 study sites from 16 countries (Supplementary Table 1). Of note, the ROPAD study was extended in 2021 with slightly modified inclusion criteria, and we have included in the present report only the study participants who were recruited in the first 26 study months (April 2019-May 2021). All study participants who were recruited to the ROPAD study within the above-mentioned period had to have age of ≥18 years at the time of enrolment and to meet at least 1 of the following criteria: (i) clinical diagnosis of PD, (ii) first- or second-degree relative of a participant positive for a pathogenic or likely pathogenic LRRK2 variant, (iii) North African Berber or Ashkenazi Jew. The latter two groups comprised 580 individuals and were not analysed in the present report. A clinical diagnosis of PD was made by experienced movement disorders specialists based on the MDS Clinical Diagnostic Criteria for Parkinson's Disease.[1] For qualifying individuals who consented to participation (by signing a written informed consent), a neurological examination was performed, medical and family histories were documented, and a dried blood spot sample was collected. The ROPAD study electronic case report form (eCRF) collects information on six main racial and ethnic categories: (i) American Indian or Alaska Native, (ii) Asian, (iii) Black or African American, (iv) Hispanic or Latino, (v) Native Hawaiian or Other Pacific Islander, (vi) White. The study is being conducted in accordance with the Declaration of Helsinki. To circumvent any potential biases, the same study protocol was closely followed at all recruitment sites and included patients with the entire range of ages at onset (AAOs) and irrespective of their family history or putative inheritance patterns. Standardized pre-test genetic counselling was offered to all ROPAD study participants by the referring neurologists. Whole post-test genetic counselling was either offered locally or by geneticists centrally supporting local principal investigators. Family history was considered positive for patients who reported being biologically related to an individual with PD, irrespective of the degree of relatedness. Although the ROPAD study in general recruits also unaffected individuals and more than one member per family, we have included only a single index patient per family in the present report. This strategy was chosen to avoid an inflation of variant carriers due to their relatedness. However, we could not control for the possibility that members of the same family, or even the same patient, were recruited by two independent centres. In addition, it was the responsibility of each recruiting centre to recruit each patient only once and to record for each newly recruited study participant any family relationship with the previously recruited patient(s).

Genetic analyses

DNA was extracted from dried blood spots on filter cards (CentoCard®) with QIASymphony using a magnetic bead-based method (Qiagen), with an acceptance criterion of minimum 3 ng/µl.

GBA1 and LRRK2 targeted sequencing

In the initially investigated subgroup of participants (n=3127; Fig. 1), the presence of 11 pathogenic (P) or likely pathogenic (LP) *LRRK2* variants was tested, and the *GBA1* coding sequence was analysed by combining a primary *GBA1*-specific long-range polymerase chain reaction (PCR) with subsequent *GBA1* exon-specific PCR and next-generation sequencing (NGS) of the resulting products as previously reported.[2,3] Of note, although we have not determined the phase of *GBA1* risk variants in individuals in whom we detected more than one such variant, we measured the glucosylsphingosine (Lyso-Gb1) biomarker in the blood of patients to exclude that they have Gaucher's disease (GD), as previously described.[4]

Gene panel analyses

Genomic DNA was enzymatically fragmented, and regions of interest were enriched using DNA capture probes (Twist Biosciences, custom design). The final indexed libraries were sequenced on an Illumina platform (NovaSeq6000, NextSeq500), with a sequencing quality parameter of 99.5% coverage of the targeted regions with a minimum read depth of 20x. The NGS panel included 50 target genes (listed in Supplementary Table 2) and was designed in 2018 shortly before the ROPAD study commenced. We included genes for which it was known at that time that their variants may cause classical PD or syndromes in which parkinsonism, even if atypical, may be a major component. Furthermore, the main genes linked to monogenic dystonia/dyskinesia and dementia, disorders with possible phenotypic overlap with PD and parkinsonism, also became part of the panel.

When employing the customized PD panel, the coding regions, 10 bp of flanking intronic sequences, and known coding and non-coding P/LP variants based on ClinVar, Human Gene Mutation Database® (HGMD), and CENTOGENE's Bio/Databank,[5] of the selected genes were targeted for analysis. Data analysis, including alignment to the hg19 human reference genome (Genome Reference Consortium GRCh37), variant calling, and annotation was performed using a validated in-house pipeline.[6] Variants with insufficient quality scores were confirmed via Sanger sequencing according to our established criteria.[7]

A semi-automated filtering strategy for the single-nucleotide variants (SNVs) and small indels was used, with the variants fulfilling the following criteria being selected for further evaluation: (i) variants previously classified in CENTOGENE's Bio/Databank as P/LP, or as variants of uncertain significance (VUS); (ii) unclassified variants; (iii) variants with minor allele frequency (MAF) < 1% including our healthy cohort, and in silico predictions of high/moderate impact on protein function; (iv) all variants described as disease-causing by external databases (HGMD and ClinVar); (v) variants with adaptive boosting (ADA) and random forest (RF) scores from dbscSNV19 > 0.6, consistent with predictions of abnormal splicing; (6) exclusion of variants previously classified as (likely) benign. Our healthy cohort includes approximately 24,000 adult individuals (>18 years) who are said to be unaffected, had no linked human phenotype ontology (HPO) terms, and had genome/exome data available.

The variants were then evaluated with respect to their pathogenicity and causality and are classified into five classes (P, LP, VUS, likely benign, benign), according to the criteria and rules for combining criteria to classify sequence variants suggested by the American College of Medical Genetics and Genomics and the Association for Molecular Pathology.[8] The 2790 unique P, LP, and VUS variants detected in the ROPAD participants were classified between July 2015 and May 2022. Namely, at the time when they were identified by our gene panel, nearly 90% of variants (n=2477) have already been present (and classified) in CENTOGENE Biodatabank, an internal database holding entries on >650000 patients that underwent genetic testing at CENTOGENE GmbH. The remaining variants were initially classified from their discovery until May 2022. Of note, in the period between July 2021 and December 2022, >30 unique variants were reclassified based on publications of novel clinical or functional evidence.

Whole-genome sequencing

For a sub-cohort of 2587 cases with no reportable genetic finding after PD panel sequencing, whole genome sequencing (WGS) was performed. In brief, DNA was extracted from dried blood spots on filter cards (CentoCard®) using standard, spin column-based methods. Genomic DNA was fragmented by sonication and Illumina adapters were ligated to generated fragments for

subsequent sequencing on the HiSeqX platform (Illumina) to yield an average coverage depth of at least 30×. An average coverage of 41× was obtained in this sample set.
Bioinformatic analysis was based on the validated pipeline published elsewhere.[9] Briefly, raw sequence data analysis, including base calling, de-multiplexing, alignment to the hg19 human reference genome (Genome Reference Consortium GRCh37), and variant calling, was performed using Dynamic Read Analysis for GENomics (DRAGEN) Bio-IT platform (Illumina, Inc., San Diego, CA). The short reads were aligned to the GRCh37 (hg19) build of the human reference genome using DRAGEN algorithm.[10] Variant calling was performed on the alignment files SNVs, and indels using DRAGEN Small Variant Caller.[11] DRAGEN[12] and Manta[13] were used for detecting SVs and CNVs. Variants were annotated using SnpEff[14] and in-house ad hoc bioinformatics tools.[6] A collection of in silico prediction tools were applied to evaluate the conservation and possible effect of the detected variants: FATHMM, PROVEAN, SIFT, PolyPhen2-HDIV, ada_score, rf_score, MutationTaster,VEST3, LRT, MutationAssessor, MetaSVM, MetaLR, MCAP, REVEL, MutPred, CADD, DANN, GERP++NR, GERP++RS, phyloP100way_vertebrate, phyloP20way_mammalian, phastCons100way_vertebrate, and phastCons20way_mammalian. All the values were fetched from the dbNSFP database.[15] The estimation of individual ancestry using genetic data is particularly valuable in disease association studies, particularly for interpreting personal genomic variation. For our analysis, we employed a tool focused on ethnicity that utilizes allele frequencies from reference populations in the 1000 Genomes Project, along with individual genotype or sequence data. This tool utilizes the BFGS optimization algorithm to accurately calculate a maximum likelihood estimate of the global admixture proportions.[16] We predicted the top three ethnicities along with their corresponding predicted values for all ROPAD WGS samples.

Supplementary Results

Pilot study: Targeted GBA1 and LRRK2 analysis

Among the 373 individuals who had a positive PD-relevant genetic test (PDGT) based on our targeted *GBA1* and *LRRK2* analyses, 288 (77·2%) patients were heterozygous for one *GBA1* risk factor (RF), while 13 (3.5%) had two heterozygous RFs, the phase of which was not determined. Out of the 45 unique *GBA1* RF variants (322 in total) identified in this ROPAD patient subgroup, the most frequent were c.1093G>A (p.Glu365Lys), c.1223C>T (p.Thr408Met), and c.1226A>G (p.Asn409Ser), found in 103, 79, and 46 individuals, respectively. Of note, unique variants refer to distinct genetic variants, while the total number of variants indicates the total number of observed variants, sometimes including multiple observations of the same unique variant (e.g., if two patients harbored the same variant, this variant would be one unique variant found two times in total). Among the 65 participants with a single *LRRK2* variant, 59 were heterozygous and two were homozygous for the c.6055G>A (p.Gly2019Ser) variant, and four harbored the heterozygous c.4321C>T (p.Arg1441Cys) variant. Seven individuals harbored a combination of one *GBA1* and one *LRRK2* variant (denoted as: *LRRK2+GBA1*), either of which would have resulted in a positive PDGT report even on its own. Thus, by targeted analysis, variants in *GBA1*, *LRRK2*, or *LRRK2+GBA1* were found in 9.6%, 2.1%, and 0.2% of the investigated 3127 patients, respectively (Supplementary Table 5).

Variants detected by gene panel sequencing

Of 12207 ROPAD participants investigated by panel sequencing, in 5361 (43.9%) patients, we found at least one P, LP, VUS, or RF) (Fig. 1, Supplementary Table 6). These individuals carried a total of 7597 variants (2790 unique, average: 1.4 (±0.7, range: 1-5) (Supplementary Fig. 4).

Findings in Parkinson's disease-related genes

Forty-six (4.5%) of the 1010 *GBA1* RF carriers who received a positive PDGT report had two heterozygous variants, the phase of which was not determined.

Forty-two (0.3%) individuals, with a positive PDGT based on variants in *GBA1*, *LRRK2*, *PRKN*, *SNCA*, or *PINK1*, had one additional heterozygous P or LP variant in one of the genes related to autosomal recessive (AR) PD (Supplementary Table 7). In *GBA1*, the three most prevalent variants were c.1093G>A (p.Glu365Lys), c.1223C>T (p.Thr408Met), and c.1226A>G (p.Asn409Ser), identified in 286, 233, and 219 of participants screened by gene panel analysis, respectively (Supplementary Table 6). Of 251 patients harbouring c.6055G>A (p.Gly2019Ser) in *LRRK2*, four individuals had this variant in a homozygous state. The fifth most frequent variant among P/LP/RF variants was c.823C>T (p.Arg275Trp) in *PRKN* found in 96 patients.

Altogether, 297 copy-number variants (CNVs) (66 unique) were found in *PRKN*, *GBA1*, *SNCA*, *PARK7*, *PINK1*, and *VPS35* (Supplementary Table 6). The highest total number of CNVs (200; 42 unique) was found in *PRKN*, while there were 17 (whole-gene amplifications) CNVs in *SNCA*, eight (five unique) CNVs in *PARK7*, and four (three unique) CNVs in *PINK1* (Supplementary Table 6). In *GBA1*, we discovered 66 (13 unique) recombinations.

Among the 1655 P/LP/RF variants in 1491 patients with a positive PDGT, 194 (51 unique) (11.7%) were CNVs, while 1471 (116 unique) (88.3%) were short sequence variants (SSVs; single-nucleotide variants and indels). Of the above-mentioned 1655 variants, only 1633 variants (189 (11.6%) CNVs and 1444 (88.4%) SSVs actually contributed to a positive PDGT of the patients (Supplementary Table 8). In summary, in *SNCA*, *PRKN*, and *PARK7*, a considerable portion of variants contributing to a positive PDGT were CNVs (Supplementary Table 8).

Only one patient with P/LP variants in two different ARPD genes was identified (Supplementary Table 9). This patient did not receive a positive PDGT report. His age at onset was 53 years and thus, comparable to the median age at onset of the 132 patients in the *PRKN*-, *PINK1*-, or *PARK7*-related PDGT-positive group (Table 1).

Findings in parkinsonism-related genes

Out of a total of 2069 variants (1004 unique) identified in genes related to atypical parkinsonism, dystonia-parkinsonism, or neurodegenerative disorders that may present with prominent or predominant parkinsonism, in the 1883 panel-screened ROPAD study participants, 37 (22 unique) were classified as P, 84 (54 unique) as LP, and 1,948 (928 unique) as VUS (Supplementary Table 6).

Forty-two study participants received a report with a positive genetic testing finding for genes related to atypical parkinsonism, dystonia-parkinsonism, or neurodegenerative disorders. Namely, 30 (0.2%) individuals had P/LP variants in *GCH1* (one CNV), and three (0.02%) and two (0.02%) patients had relevant variants in *PLA2G6* and *PDGFRB* respectively (Table 3, Supplementary Table 7). CNVs (exonic/whole gene deletions and duplications) were identified in *ATP1A3, DNAJC6, GCH1, PLA2G6, SLC30A10,* and *SLC6A3*, we identified only two large deletions encompassing several exons of *MCOLN1* and *SLC19A3* (Supplementary Table 6).

Findings in dystonia/dyskinesia-related genes

Out of a total of 1,639 variants (857 unique) identified in genes related to dystonia/dyskinesia in the 1,505 panel-screened ROPAD study participants, 72 (24 unique) were classified as P, 57 (41 unique) as LP, and 1,507 (792 unique) as VUS (Supplementary Table 6). One variant (p.Arg288* in *TOR1A* found in three patients) was scored as VUS with respect to DYT-*TOR1A* and LP with respect to arthrogryposis multiplex congenita 5 (OMIM# 618947).

Twenty-six study participants received a report with a positive genetic testing finding for dystonia/dyskinesia-related genes. Namely, 12 (0.1%) individuals had P/LP variants in *TOR1A* (half of those were c.907_909delGAG), and eight (0.06%), four (0.03%), one (0.01%), and one (0.01%) patient had relevant variants in *SGCE* (three CNVs), *GNAL* (one CNV), *THAP1*, and *KMT2B*, respectively (Table 3, Supplementary Table 7). Apart from the four CNVs in *SGCE* and *GNAL*, we identified only two large deletions encompassing several exons of *MCOLN1* and *SLC19A3* (Supplementary Table 6). All other changes were SSVs and all but two were heterozygous (Supplementary Table 6).

Findings in dementia-related genes

The seven dementia-related genes present in our panel harboured 732 variants (302 unique) present in 695 of our patients (Supplementary Table 4). Most of the variants (699 variants; 279 unique) were classified as VUS, whereas 9 variants (7 unique) were LP, and 24 (16 unique) were P (Supplementary Table 6).

Thirty patients received a report with a positive genetic testing finding for *GRN* (n=19), *MAPT* (n=5), *PSEN1* (n=2), *PSEN2* (n=2), and *APP* (n=2) (Table 3 and Supplementary Table 7). No CNVs were detected in dementia-related genes in the ROPAD Study patients.

Variant reclassification

In the period between July 2021 and December 2022, 32 unique variants were reclassified in 59 patients based on publications of novel clinical or functional evidence. Eleven of those variants found in 42 patients were reclassified from VUS (variant of uncertain significance) into P/LP and 4 variants (found in 10 patients) from VUS to P/LP. Due to our reclassification efforts, 32 individuals received a positive PDGT report.

Supplementary Discussion

More than two-thirds of the ROPAD patients were recruited at tertiary referral centres, which may have led to a slight overestimation of the genetic contribution although the mean AAO or percentage of patients with a positive family history did not seem to differ considerably from those in even more unselected samples.

The median AAOs related to major PD genetic forms in ROPAD recapitulated the trends described in the literature but were in some instances slightly higher than previously reported (ROPAD vs. literature: *PRKN*/*PINK1/PARK7*: 35 vs. 31 years, *SNCA*: 50 vs. 46 years, *LRRK2*: 58 vs. 57 years, *GBA1*: 56 vs. 57 years).[17–19]

The finding of almost identical numbers of CNVs and SSVs in patients with *PRKN*-related PD and twice more CNVs than SSVs in *SNCA*-related PD, underlines the necessity of quantitative screening methods in the diagnosis of these genetic PD forms.

The p.Glu365Lys and p.Thr408Met *GBA1* variants were detected with significantly higher frequency in ROPAD patients in comparison to the Genome Aggregation Database (gnomAD) overall or the European (EUR) population (p.Glu365Lys: ROPAD: 0.0154, gnomAD (all): 0.0105, gnomAD (EUR): 0.0121; p.Thr408Met: ROPAD: 0.0123, gnomAD (all): 0.0059, gnomAD (EUR): 0.0092; $P<0.00001$).

Supplementary References

1. Postuma RB, Berg D, Stern M, et al. MDS clinical diagnostic criteria for Parkinson’s disease. *Mov Disord*. 2015;30(12):1591-1601. doi:10.1002/MDS.26424
2. Skrahina V, Gaber H, Vollstedt EJ, et al. The Rostock International Parkinson’s Disease (ROPAD) Study: Protocol and Initial Findings. *Mov Disord*. 2021;36(4):1005-1010. doi:10.1002/MDS.28416
3. Cullufi P, Tabaku M, Beetz C, et al. Comprehensive clinical, biochemical and genetic screening reveals four distinct GBA genotypes as underlying variable manifestation of Gaucher disease in a single family. *Mol Genet Metab reports*. 2019;21:100532. doi:10.1016/j.ymgmr.2019.100532
4. Rolfs A, Giese A-K, Grittner U, et al. Glucosylsphingosine is a highly sensitive and specific biomarker for primary diagnostic and follow-up monitoring in Gaucher disease in a non-Jewish, Caucasian cohort of Gaucher disease patients. Dardis A, ed. *PLoS One*. 2013;8(11):e79732. doi:10.1371/journal.pone.0079732
5. Trujillano D, Oprea G-E, Schmitz Y, Bertoli-Avella AM, Abou Jamra R, Rolfs A. A comprehensive global genotype-phenotype database for rare diseases. *Mol Genet genomic Med*. 2017;5(1):66-75. doi:10.1002/mgg3.262
6. Trujillano D, Bertoli-Avella AM, Kumar Kandaswamy K, et al. Clinical exome sequencing: results from 2819 samples reflecting 1000 families. *Eur J Hum Genet*. 2017;25(2):176-182. doi:10.1038/ejhg.2016.146
7. Bauer P, Kandaswamy KK, Weiss MER, et al. Development of an evidence-based algorithm that optimizes sensitivity and specificity in ES-based diagnostics of a clinically heterogeneous patient population. *Genet Med*. 2019;21(1):53-61. doi:10.1038/s41436-018-0016-6
8. Richards S, Aziz N, Bale S, et al. Standards and guidelines for the interpretation of sequence variants: a joint consensus recommendation of the American College of Medical Genetics and Genomics and the Association for Molecular Pathology. *Genet Med*. 2015;17(5):405-423. doi:10.1038/gim.2015.30
9. Bertoli-Avella AM, Beetz C, Ameziane N, et al. Successful application of genome sequencing in a diagnostic setting: 1007 index cases from a clinically heterogeneous cohort. *Eur J Hum Genet*. 2021;29(1):141-153. doi:10.1038/s41431-020-00713-9
10. Raczy C, Petrovski R, Saunders CT, et al. Isaac: ultra-fast whole-genome secondary analysis on Illumina sequencing platforms. *Bioinformatics*. 2013;29(16):2041-2043. doi:10.1093/BIOINFORMATICS/BTT314
11. Miller NA, Farrow EG, Gibson M, et al. A 26-hour system of highly sensitive whole genome sequencing for emergency management of genetic diseases. *Genome Med*. 2015;7(1). doi:10.1186/S13073-015-0221-8
12. Roller E, Ivakhno S, Lee S, Royce T, Tanner S. Canvas: Versatile and scalable detection of copy number variants. *Bioinformatics*. 2016;32(15):2375-2377. doi:10.1093/bioinformatics/btw163
13. Chen X, Schulz-Trieglaff O, Shaw R, et al. Manta: rapid detection of structural variants and indels for germline and cancer sequencing applications. *Bioinformatics*. 2016;32(8):1220-1222. doi:10.1093/BIOINFORMATICS/BTV710
14. Cingolani P, Platts A, Wang LL, et al. A program for annotating and predicting the effects of single nucleotide polymorphisms, SnpEff: SNPs in the genome of Drosophila melanogaster strain w1118; iso-2; iso-3. *Fly (Austin)*. 2012;6(2):80-92. doi:10.4161/FLY.19695
15. Liu X, Wu C, Li C, Boerwinkle E. dbNSFP v3.0: A One-Stop Database of Functional Predictions and Annotations for Human Nonsynonymous and Splice-Site SNVs. *Hum Mutat*. 2016;37(3):235-241. doi:10.1002/HUMU.22932
16. Bansal V, Libiger O. Fast individual ancestry inference from DNA sequence data leveraging allele frequencies for multiple populations. *BMC Bioinformatics*. 2015;16(1). doi:10.1186/S12859-014-0418-7
17. Trinh J, Zeldenrust FMJ, Huang J, et al. Genotype-phenotype relations for the Parkinson’s disease genes SNCA, LRRK2, VPS35: MDSGene systematic review. *Mov Disord*. 2018;33(12):1857-1870. doi:10.1002/mds.27527
18. Kasten M, Hartmann C, Hampf J, et al. Genotype-Phenotype Relations for the Parkinson’s Disease Genes Parkin, PINK1, DJ1: MDSGene Systematic Review. *Mov Disord*. 2018;33(5). doi:10.1002/mds.27352
19. Olszewska DA, McCarthy A, Soto-Beasley AI, et al. Association Between Glucocerebrosidase Mutations and Parkinson’s Disease in Ireland. *Front Neurol*. 2020;11:527. doi:10.3389/FNEUR.2020.00527/FULL

Supplementary Tables

Supplementary Table 1A. Demographic data of the ROPAD patient cohort and different genetic subgroups

			All study participants (n=12580)	PDGT-positive group (patients with a positive PDGT) (n=1864)	Idiopathic group (patients without P/LP/VUS variants identified by gene panel sequencing) (n=6846)	*GBA1*-related PDGT-positive group (n=1311)
Recruitment centre country	Europe	Albania	37	7	27	6
		Belgium	126	21	60	15
		France	13	3	5	3
		Germany	2999	449	1724	378
		Greece	36	4	24	3
		Italy	1089	131	578	97
		Norway	229	43	151	34
		Portugal	10	1	2	1
		Spain	747	136	334	61
		United Kingdom	540	82	333	56
	Middle East	Israel	1315	257	592	138
		Turkey	989	129	539	75
	North America	Canada	13	3	4	1
		United States	3360	476	1970	360
	South America	Argentina	121	16	60	12
		Brazil	956	106	443	71
Patient country		Albania	27	4	21	4
		Argentina	40	6	19	3
		Belgium	43	8	22	5
		Brazil	213	21	114	13
		Germany	1784	260	1098	222
		Greece	28	3	20	2
		Israel	419	90	201	50
		Italy	405	51	237	36
		Norway	229	43	151	34
		Spain	207	40	105	8
		Turkey	559	66	372	37
		United Kingdom	256	37	195	29
		United States	3002	412	1809	315
		Unknown	5368	823	2482	553

PD: Parkinson's disease; PDGT: PD-relevant genetic test; P/LP/VUS: pathogenic/likely pathogenic/variant with uncertain significance; IQR: interquartile range.

Supplementary Table 1A. Continued

			***LRRK2*-related PDGT-positive group (n=368)**	***LRRK2+GBA1*-related PDGT-positive group (n=23)**	***PRKN*-, *PINK1*-, or *PARK7* related PDGT-positive group (n=132)**	***SNCA*-related PDGT-positive group (n=25)**
Recruitment centre country	Europe	Albania	0	0	1	0
		Belgium	4	0	2	0
		France	0	0	0	0
		Germany	34	6	26	3
		Greece	0	0	0	1
		Italy	18	0	11	5
		Norway	7	1	1	0
		Portugal	0	0	0	0
		Spain	63	1	11	0
		United Kingdom	16	0	6	3
	Middle East	Israel	97	8	13	1
		Turkey	14	0	32	6
	North America	Canada	0	0	2	0
		United States	90	6	15	5
	South America	Argentina	3	0	1	0
		Brazil	22	1	11	1
Patient country		Albania	0	0	0	0
		Argentina	2	0	1	0
		Belgium	2	0	1	0
		Brazil	6	0	2	0
		Germany	19	1	16	1
		Greece	0	0	0	1
		Israel	33	3	4	0
		Italy	6	0	7	2
		Norway	7	1	1	0
		Spain	25	1	6	0
		Turkey	5	0	18	5
		United Kingdom	5	0	1	2
		United States	73	6	14	4
		Unknown	185	11	61	10

PD: Parkinson's disease; PDGT: PD-relevant genetic test; P/LP/VUS: pathogenic/likely pathogenic/variant with uncertain significance; IQR: interquartile range.

Supplementary Table 1B. Percentage of patients who received a Parkinson's disease-relevant positive genetic testing report out of all initially recruited patients

		Percentage of patients with a positive PDGT out of initially recruited patients in respective countries (%)
Recruitment centre country	Albania	18.9
	Argentina	13.2
	Belgium	16.7
	Brazil	11.1
	Canada	23.1
	France	23.1
	Germany	15.0
	Greece	11.1
	Israel	19.5
	Italy	12.0
	Norway	18.8
	Portugal	10.0
	Spain	18.2
	Turkey	13.0
	United Kingdom	15.2
	United States	14.2
	Overall	14.8

PD: Parkinson's disease; PDGT: PD-relevant genetic testing

Supplementary Table 2. Overview of the genes investigated in the ROPAD study participants and their relevant transcripts, related phenotypes, and inheritance patterns

Gene	Relevant transcript[a]	Related phenotype	Inheritance pattern
CHCHD2	NM_016139.2	Parkinson's disease	Autosomal dominant
GBA1	NM_000157.4		Autosomal dominant
LRRK2	NM_198578.4		Autosomal dominant
PARK7	NM_007262.5		Autosomal recessive
PINK1	NM_032409.3		Autosomal recessive
PRKN	NM_004562.3		Autosomal recessive
SNCA	NM_000345.4		Autosomal dominant
VPS35	NM_018206.6		Autosomal dominant
ATP13A2	NM_022089.4	Atypical parkinsonism	Autosomal recessive
DCTN1	NM_004082.5		Autosomal dominant
DNAJC6	NM_001256864.2		Autosomal recessive
FBXO7	NM_012179.4		Autosomal recessive
RAB39B	NM_171998.4		X-linked
SYNJ1	NM_203446.2		Autosomal recessive
VPS13C	NM_020821.3		Autosomal recessive
ATP1A3	NM_152296.5	Dystonia-parkinsonism	Autosomal dominant
C19orf12	NM_001031726.2		Autosomal dominant/recessive
GCH1	NM_000161.3		Autosomal dominant
PLA2G6	NM_003560.4		Autosomal recessive
SLC30A10	NM_018713.3		Autosomal recessive
SLC6A3	NM_001044.5		Autosomal recessive
PDE8B	NM_003719.5	Neurodegenerative disorders that may include (atypical) parkinsonism	Autosomal dominant
PDGFB	NM_002608.4		Autosomal dominant
PDGFRB	NM_002609.4		Autosomal dominant
SLC20A2	NM_001257180.2		Autosomal dominant
XPR1	NM_004736.4		Autosomal dominant
ADCY5	NM_183357.2	Dystonia/dyskinesia	Autosomal dominant
ANO3	NM_031418.2		Autosomal dominant
COX20	NM_198076.6		Autosomal recessive
GCDH	NM_000159.4		Autosomal recessive
GNAL	NM_001142339		Autosomal dominant
HPCA	NM_002143.3		Autosomal recessive
KCTD17	NM_001282684.2		Autosomal dominant
KMT2B	NM_014727.3		Autosomal dominant
MCOLN1	NM_020533.3		Autosomal recessive
PANK2	NM_153638.4		Autosomal recessive
PRKRA	NM_003690.5		Autosomal recessive
SGCE	NM_003919.3		Autosomal dominant
SLC19A3	NM_025243.4		Autosomal recessive
SLC39A14	NM_015359.6		Autosomal recessive
TAF1	NM_001286074.1		X-linked
THAP1	NM_018105.3		Autosomal dominant
TOR1A	NM_000113.3		Autosomal dominant
VAC14	NM_018052.3		Autosomal recessive
APOE	NM_000041.4	Dementia	Autosomal dominant
APP	NM_000484.3		Autosomal dominant
GRN	NM_002087.4		Autosomal dominant
MAPT	NM_001123066.3		Autosomal dominant/recessive
PSEN1	NM_000021.4		Autosomal dominant
PSEN2	NM_000447.3		Autosomal dominant

[a]Transcripts relevant for the respective disorders, i.e., not necessarily the canonical transcripts of the respective genes

Supplementary Table 3. Overview of patient ethnicities in each recruitment centre country

A. For all study participants

	All study participants (n=12580)								
**Recruitment center country**	**White**	**Asian**	**Hispanic or Latino**	**Black or African American**	**Native Hawaiian or Other Pacific Islander**	**American Indian or Alaska Native**	**Other**	**Unknown**	**Sum**
Albania	37	0	0	0	0	0	0	0	37
Argentina	52	1	0	0	0	0	68	0	121
Belgium	124	0	0	1	0	0	1	0	126
Brazil	706	23	22	89	0	5	111	0	956
Canada	11	2	0	0	0	0	0	0	13
France	12	1	0	0	0	0	0	0	13
Germany	2983	6	1	2	1	1	5	0	2999
Greece	35	0	0	0	0	0	1	0	36
Israel	962	2	0	0	0	0	350	1	1315
Italy	1084	3	0	1	0	1	0	0	1089
Norway	227	0	0	0	0	0	0	2	229
Portugal	10	0	0	0	0	0	0	0	10
Spain	711	1	0	2	0	17	11	5	747
Turkey	989	0	0	0	0	0	0	0	989
United Kingdom	522	12	0	1	0	1	4	0	540
United States	3114	88	26	53	4	7	68	0	3360
Sum	11579	139	49	149	5	32	619	8	12580

B. For patients with a positive Parkinson's disease-relevant genetic test (PDGT)

	PDGT-positive group (patients with a positive PDGT) (n=1864)								
**Recruitment center country**	**White**	**Asian**	**Hispanic or Latino**	**Black or African American**	**Native Hawaiian or Other Pacific Islander**	**American Indian or Alaska Native**	**Other**	**Unknown**	**Sum**
Albania	7	0	0	0	0	0	0	0	7
Argentina	5	1	0	0	0	0	10	0	16
Belgium	20	0	0	0	0	0	1	0	21
Brazil	78	2	1	8	0	0	17	0	106
Canada	1	2	0	0	0	0	0	0	3
France	3	0	0	0	0	0	0	0	3
Germany	447	1	0	0	0	0	1	0	449
Greece	4	0	0	0	0	0	0	0	4
Israel	175	0	0	0	0	0	82	0	257
Italy	129	1	0	0	0	1	0	0	131
Norway	42	0	0	0	0	0	0	1	43
Portugal	1	0	0	0	0	0	0	0	1
Spain	126	1	0	1	0	2	5	1	136
Turkey	129	0	0	0	0	0	0	0	129
United Kingdom	80	1	0	0	0	0	1	0	82
United States	450	11	3	7	0	0	5	0	476
Sum	1697	20	4	16	0	3	122	2	1864

C. For patients from the idiopathic Parkinson's disease group

	Idiopathic Parkinson's disease group (n=6846)								
**Recruitment centre country**	**White**	**Asian**	**Hispanic or Latino**	**Black or African American**	**Native Hawaiian or Other Pacific Islander**	**American Indian or Alaska Native**	**Other**	**Unknown**	**Sum**
Albania	27	0	0	0	0	0	0	0	27
Argentina	24	0	0	0	0	0	36	0	60
Belgium	60	0	0	0	0	0	0	0	60
Brazil	332	3	12	47	0	3	46	0	443
Canada	4	0	0	0	0	0	0	0	4
France	4	1	0	0	0	0	0	0	5
Germany	1714	3	0	2	1	0	4	0	1724
Greece	23	0	0	0	0	0	1	0	24
Israel	439	1	0	0	0	0	152	0	592
Italy	576	1	0	1	0	0	0	0	578
Norway	150	0	0	0	0	0	0	1	151
Portugal	2	0	0	0	0	0	0	0	2
Spain	323	0	0	1	0	6	2	2	334
Turkey	539	0	0	0	0	0	0	0	539
United Kingdom	325	4	0	1	0	0	3	0	333
United States	1827	50	22	27	3	4	37	0	1970
Sum	6369	63	34	79	4	13	281	3	6846

D. For patients from the *GBA1*-related Parkinson's disease-relevant genetic test (PDGT)-positive group

	GBA1-related PDGT-positive group (n=1311)								
**Recruitment centre country**	**White**	**Asian**	**Hispanic or Latino**	**Black or African American**	**Native Hawaiian or Other Pacific Islander**	**American Indian or Alaska Native**	**Other**	**Unknown**	**Sum**
Albania	6	0	0	0	0	0	0	0	6
Argentina	2	1	0	0	0	0	9	0	12
Belgium	15	0	0	0	0	0	0	0	15
Brazil	54	0	1	6	0	0	10	0	71
Canada	1	0	0	0	0	0	0	0	1
France	3	0	0	0	0	0	0	0	3
Germany	378	0	0	0	0	0	0	0	378
Greece	3	0	0	0	0	0	0	0	3
Israel	92	0	0	0	0	0	46	0	138
Italy	95	1	0	0	0	1	0	0	97
Norway	33	0	0	0	0	0	0	1	34
Portugal	1	0	0	0	0	0	0	0	1
Spain	59	0	0	1	0	1	0	0	61
Turkey	75	0	0	0	0	0	0	0	75
United Kingdom	55	0	0	0	0	0	1	0	56
United States	340	7	1	7	0	0	5	0	360
Sum	1212	9	2	14	0	2	71	1	1311

E. For patients from the *LRRK2*-related Parkinson's disease-relevant genetic testing (PDGT)-positive group

	LRRK2-related PDGT-positive group (n=368)								
**Recruitment centre country**	**White**	**Asian**	**Hispanic or Latino**	**Black or African American**	**Native Hawaiian or Other Pacific Islander**	**American Indian or Alaska Native**	**Other**	**Unknown**	**Sum**
Albania	0	0	0	0	0	0	0	0	0
Argentina	3	0	0	0	0	0	0	0	3
Belgium	4	0	0	0	0	0	0	0	4
Brazil	16	2	0	1	0	0	3	0	22
Canada	0	0	0	0	0	0	0	0	0
France	0	0	0	0	0	0	0	0	0
Germany	32	1	0	0	0	0	1	0	34
Greece	0	0	0	0	0	0	0	0	0
Israel	68	0	0	0	0	0	29	0	97
Italy	18	0	0	0	0	0	0	0	18
Norway	7	0	0	0	0	0	0	0	7
Portugal	0	0	0	0	0	0	0	0	0
Spain	56	1	0	0	0	1	4	1	63
Turkey	14	0	0	0	0	0	0	0	14
United Kingdom	15	1	0	0	0	0	0	0	16
United States	86	3	1	0	0	0	0	0	90
Sum	319	8	1	1	0	1	37	1	368

F. For patients from the *LRRK2+GBA1*-related Parkinson's disease-relevant genetic test (PDGT)-positive group

	***LRRK2+GBA1*-related PDGT-positive group (n=23)**								
**Recruitment centre country**	**White**	**Asian**	**Hispanic or Latino**	**Black or African American**	**Native Hawaiian or Other Pacific Islander**	**American Indian or Alaska Native**	**Other**	**Unknown**	**Sum**
Albania	0	0	0	0	0	0	0	0	0
Argentina	0	0	0	0	0	0	0	0	0
Belgium	0	0	0	0	0	0	0	0	0
Brazil	1	0	0	0	0	0	0	0	1
Canada	0	0	0	0	0	0	0	0	0
France	0	0	0	0	0	0	0	0	0
Germany	6	0	0	0	0	0	0	0	6
Greece	0	0	0	0	0	0	0	0	0
Israel	4	0	0	0	0	0	4	0	8
Italy	0	0	0	0	0	0	0	0	0
Norway	1	0	0	0	0	0	0	0	1
Portugal	0	0	0	0	0	0	0	0	0
Spain	1	0	0	0	0	0	0	0	1
Turkey	0	0	0	0	0	0	0	0	0
United Kingdom	0	0	0	0	0	0	0	0	0
United States	6	0	0	0	0	0	0	0	6
Sum	19	0	0	0	0	0	4	0	23

G. For patients from the *PRKN-*, *PINK1-*, or *PARK7*-related Parkinson's disease-relevant genetic test (PDGT)-positive group

	***PRKN-, PINK1-, or PARK7*-related PDGT-positive group (n=132)**								
**Recruitment centre country**	**White**	**Asian**	**Hispanic or Latino**	**Black or African American**	**Native Hawaiian or Other Pacific Islander**	**American Indian or Alaska Native**	**Other**	**Unknown**	**Sum**
Albania	1	0	0	0	0	0	0	0	1
Argentina	0	0	0	0	0	0	1	0	1
Belgium	1	0	0	0	0	0	1	0	2
Brazil	6	0	0	1	0	0	4	0	11
Canada	0	2	0	0	0	0	0	0	2
France	0	0	0	0	0	0	0	0	0
Germany	26	0	0	0	0	0	0	0	26
Greece	0	0	0	0	0	0	0	0	0
Israel	11	0	0	0	0	0	2	0	13
Italy	11	0	0	0	0	0	0	0	11
Norway	1	0	0	0	0	0	0	0	1
Portugal	0	0	0	0	0	0	0	0	0
Spain	10	0	0	0	0	0	1	0	11
Turkey	32	0	0	0	0	0	0	0	32
United Kingdom	6	0	0	0	0	0	0	0	6
United States	13	1	1	0	0	0	0	0	15
Sum	118	3	1	1	0	0	9	0	132

H. For patients from the *SNCA*-related Parkinson's disease-relevant genetic test (PDGT)-positive group

	SNCA-related PDGT-positive group (n=25)								
**Recruitment centre country**	**White**	**Asian**	**Hispanic or Latino**	**Black or African American**	**Native Hawaiian or Other Pacific Islander**	**American Indian or Alaska Native**	**Other**	**Unknown**	**Sum**
Albania	0	0	0	0	0	0	0	0	0
Argentina	0	0	0	0	0	0	0	0	0
Belgium	0	0	0	0	0	0	0	0	0
Brazil	1	0	0	0	0	0	0	0	1
Canada	0	0	0	0	0	0	0	0	0
France	0	0	0	0	0	0	0	0	0
Germany	3	0	0	0	0	0	0	0	3
Greece	1	0	0	0	0	0	0	0	1
Israel	1	0	0	0	0	0	1	0	2
Italy	5	0	0	0	0	0	0	0	5
Norway	0	0	0	0	0	0	0	0	0
Portugal	0	0	0	0	0	0	0	0	0
Spain	0	0	0	0	0	0	0	0	0
Turkey	6	0	0	0	0	0	0	0	6
United Kingdom	3	0	0	0	0	0	0	0	3
United States	4	0	0	0	0	0	0	0	4
Sum	24	0	0	0	0	0	1	0	25

Supplementary Table 4. Overview of the variants detected in the 373 individuals with a PD-relevant genetic test (PDGT) based on the targeted *GBA1* and *LRRK2* analyses

Gene	Variant (cDNA level)	Variant (protein level)	Zygosity	Number of patients
GBA1	c.115+1G>A	unknown	heterozygous	5
	c.256C>T	p.Arg86*	heterozygous	1
	c.371T>C	p.Met124Thr	heterozygous	1
	c.413del	p.Pro138Leufs*62	heterozygous	1
	c.475C>T	p.Arg159Trp	heterozygous	7
	c.476G>A	p.Arg159Gln	heterozygous	1
	c.485T>C	p.Met162Thr	heterozygous	1
	c.508C>T	p.Arg170Cys	heterozygous	1
	c.509G>A	p.Arg170His	heterozygous	1
	c.535G>C	p.Asp179His	heterozygous	1
	c.604C>T	p.Arg202*	heterozygous	1
	c.635C>G	p.Ser212*	heterozygous	1
	c.667T>C	p.Trp223Arg	heterozygous	2
	c.680_681delinsGG	p.Asn227Arg	heterozygous	1
	c.680A>G	p.Asn227Ser	heterozygous	1
	c.681T>G	p.Asn227Lys	heterozygous	1
	c.701G>A	p.Gly234Glu	heterozygous	1
	c.721G>A	p.Gly241Arg	heterozygous	2
	c.762-2A>G	unknown	heterozygous	1
	c.84dupG	Leu29Alafs*18	heterozygous	3
	c.886C>T	p.Arg296*	heterozygous	1
	c.888dup	p.Asp297Argfs*6	heterozygous	1
	c.914del	p.Pro305Leufs*31	heterozygous	1
	c.928A>G	p.Ser310Gly	heterozygous	1
	c.980_982dup	p.Leu327dup	heterozygous	1
	c.1060G>C	p.Asp354His	heterozygous	1
	c.1084del	p.Thr362Profs*2	heterozygous	1
	c.1093G>A	p.Glu365Lys	heterozygous	92
			homozygous	1
	c.1174C>T	p.Arg392Trp	heterozygous	2
	c.1193G>A	p.Arg398Gln	heterozygous	1
	c.1223C>T	p.Thr408Met	heterozygous	73
			homozygous	1
	c.1226A>G	p.Asn409Ser	heterozygous	36
	c.1279G>A	p.Glu427Lys	heterozygous	7
	c.1296G>A	p.Trp432*	heterozygous	2
	c.1323C>G	p.Ile441Met	heterozygous	1
	c.1342G>C	p.Asp448His	heterozygous	6
	c.1448T>C	p.Leu483Pro	heterozygous	18
	c.1448T>G	p.Leu483Arg	heterozygous	1
	c.1495G>A	p.Val499Met	heterozygous	1
	c.1504C>T	p.Arg502Cys	heterozygous	2
	c.1505G>A	p.Arg502His	heterozygous	1
	c.1604G>A	p.Arg535His	heterozygous	1
	GBA-EX.3-12 rec	unknown	heterozygous	1

Supplementary Table 4. Continued

Gene	Variant (cDNA level)	Variant (protein level)	Zygosity	Number of patients
LRRK2	c.6055G>A	p.Gly2019Ser	heterozygous	59
			homozygous	2
	c.4321C>T	p.Arg1441Cys	heterozygous	4
GBA1	c.1448T>C	p.Leu483Pro	heterozygous	1
	GBA-EX.11-12 rec	unknown	heterozygous	
GBA1	c.1342G>C	p.Asp448His	heterozygous	1
	c.1448T>C	p.Leu483Pro	heterozygous	
	GBA-EX.10-12 rec	unknown	heterozygous	
GBA1	c.535G>C	p.Asp179His	heterozygous	5
	c.1093G>A	p.Glu365Lys	heterozygous	
GBA1	c.1223C>T	p.Thr408Met	heterozygous	1
	c.1604G>A	p.Arg535His	heterozygous	
GBA1	c.1093G>A	p.Glu365Lys	heterozygous	1
	c.1342G>C	p.Asp448His	heterozygous	
GBA1	c.1448T>C	p.Leu483Pro	heterozygous	1
	GBA-EX.11-12 rec	unknown	heterozygous	
GBA1	c.1223C>T	p.Thr408Met	heterozygous	1
	c.1226A>G	p.Asn409Ser	heterozygous	
GBA1	c.1093G>A	p.Glu365Lys	heterozygous	1
	c.1226A>G	p.Asn409Ser	heterozygous	
GBA1	c.1093G>A	p.Glu365Lys	heterozygous	1
	c.1448T>C	p.Leu483Pro	heterozygous	
GBA1	c.475C>T	p.Arg159Trp	heterozygous	1
LRRK2	c.6055G>A	p.Gly2019Ser	heterozygous	
GBA1	c.1223C>T	p.Thr408Met	heterozygous	2
LRRK2	c.6055G>A	p.Gly2019Ser	heterozygous	
GBA1	c.1226A>G	p.Asn409Ser	heterozygous	1
LRRK2	c.6055G>A	p.Gly2019Ser	heterozygous	
GBA1	c.1093G>A	p.Glu365Lys	heterozygous	2
LRRK2	c.6055G>A	p.Gly2019Ser	heterozygous	
GBA1	c.1223C>T	p.Thr408Met	heterozygous	1
LRRK2	c.4321C>T	p.Arg1441Cys	heterozygous	

Supplementary Table 5. Overview of variants in the ROPAD study participants with a positive Parkinson's disease-relevant genetic test (PDGT) based on *GBA1*- and *LRRK2*-targeted analysis

Gene to which a PGT finding was related	Number of patients with a positive PDGT in respective genes	Percentage of patients tested by GBA1/LRRK2-targeted analysis	Percentage of patients tested by *GBA1*/*LRRK2*-targeted analysis who received a PDGT report
GBA1 (heterozygous)	286	9.15	76.68
GBA1 (heterozygous) +GBA1 (heterozygous)	13	0.42	3.49
GBA1 (homozygous)	2	0.06	0.54
Sum GBA1:	**301**	**9.63**	**80.7**
LRRK2 (heterozygous)	63	2.01	16.89
LRRK2 (homozygous)	2	0.06	0.54
Sum LRRK2:	**65**	**2.08**	**17.43**
GBA1 (heterozygous) +LRRK2 (heterozygous)	7	0.22	1.88
Sum (targeted GBA1/LRRK2 analysis)	**373**	**11.93**	**100**

PDGT: Parkinson's disease-relevant genetic test

Supplementary Table 7. Overview of variants in the study participants with a positive PD-relevant genetic test (PDGT) based on gene panel analysis

**Gene to which a PGT finding was related**	**Number of patients with a positive PDGT finding in respective genes**	**Percentage of patients tested by gene panel analysis**	**Percentage of patients tested by gene panel sequencing who had a positive PDGT**
GBA1	921	7.54	61.77
GBA1+GBA1	44	0.36	2.95
GBA1 (homozygous)	5	0.04	0.34
GBA1+AR PD gene	30	0.25	2.01
GBA1+MAPT	1	0.01	0.07
GBA1+TOR1	3	0.02	0.20
GBA1+GBA1+AR PD gene	1	0.01	0.07
GBA1+GBA1+GNAL	1	0.01	0.07
Sum GBA1:	**1006**	**8.24**	**67.47**
LRRK2	292	2.39	19.58
LRRK2 (homozygous)	3	0.02	0.20
LRRK2+AR PD gene	6	0.05	0.40
LRRK2 (homozygous)+AR PD gene	1	0.01	0.07
LRRK2+APP	1	0.01	0.07
Sum LRRK2	**303**	**2.48**	**20.32**
PRKN (homozygous or two heterozygous variants)	117	0.96	7.85
PRKN (homozygous or two heterozygous variants)+AR PD gene	1	0.01	0.07
Sum PRKN	**118**	**0.97**	**7.91**
SNCA	20	0.16	1.34
SNCA (homozygous)	3	0.02	0.20
SNCA+AR PD gene	2	0.02	0.13
Sum SNCA	**25**	**0.2**	**1.68**
PINK1 (homozygous or two heterozygous variants)	8	0.07	0.54
PINK1 (homozygous or two heterozygous variants)+AR PD gene	1	0.01	0.07
Sum PINK1	**9**	**0.07**	**0.60**
PARK7 (homozygous or two heterozygous variants)	3	0.02	0.20
PARK7 (homozygous or two heterozygous variants)+GRN	1	0.01	0.07
Sum PARK7	**4**	**0.03**	**0.27**
VPS35	2	0.02	0.13
GBA1+LRRK2	16	0.13	1.07
GBA1+GCH1	4	0.03	0.27
GBA1+PRKN (homozygous or two heterozygous variants)	2	0.02	0.13
GBA1+VPS35	1	0.01	0.07
PRKN+GCH1	1	0.01	0.07
Sum (panel-based positive PDGT)	**1491**	**12.21**	**100.00**

Unless stated otherwise (e.g., homozygous), a gene name (or AD PD or AR PD) denotes a single heterozygous pathogenic or likely pathogenic variant in a gene other than GBA. GBA denotes a single heterozygous risk factor. PGT: positive genetic testing; PDGT: Parkinson's disease-relevant genetic test; AD PD gene: single heterozygous pathogenic (P) or likely pathogenic (LP) variant in one autosomal-dominant Parkinson's disease-related gene; AR PD gene: single heterozygous pathogenic or likely pathogenic variant in one autosomal-recessive Parkinson's disease-related gene

Supplementary Table 8. Overview of the variants that contributed to a positive Parkinson's disease-relevant genetic test (PDGT) report

		**Pathogenic/likely pathogenic/risk factor variants contributing to a positive PDGT report**				
**Gene**	**Number of patients with a positive PDGT in respective genes**	**Number of variants**	**Copy number variants (CNVs)**	**Short sequence variants (SSVs)**	**Homozygous**	**Heterozygous**
GBA1	1010	1056	66	990	5	1051
LRRK2	303	303	0	303	4	299
PRKN	119	193	101	92	55	138
SNCA	25	25	17	8	4	21
PINK1	9	11	1	10	7	4
PARK7	4	4	2	2	4	0
VPS35	2	2	0	2	0	2
GBA1+LRRK2	16	32	0	32	0	32
GBA1+PRKN	2	5	2	3	1	4
GBA1+VPS35	1	2	0	2	0	2
Sum:	1491	1633*	189 (11.6%)	1444 (88.4%)	80	1553

*The 32 single heterozygous variants in autosomal recessive Parkinson's disease genes that were found in some of these samples but did not contribute to a positive PDGT report, per se, were not considered here. Thus, this number does not amount to 1665. PDGT: Parkinson's disease-related genetic testing

Supplementary Table 9. Overview of variants in the ROPAD study participants with variants in the PD-related genes who did not receive a positive Parkinson's disease-relevant genetic test (PDGT) report

Gene panel finding	Number of patients	Number of variants
Single heterozygous P/LP variant in *PRKN*	211	211
Single heterozygous P/LP variant in *PINK1*	40	40
Single heterozygous P/LP variant in *PARK7*	7	7
Single heterozygous P/LP variant + single heterozygous VUS in *PRKN*	7	14
Single heterozygous P/LP variant + single heterozygous VUS in *PINK1*	1	2
Single heterozygous P/LP variant in *PINK1* + single heterozygous P/LP variant in *PARK7*	1	2
Single heterozygous P/LP variant in an AR PD gene + one VUS in an AD PD gene	16	32
Single heterozygous P/LP variant in an AR PD gene + one VUS in an AR PD gene	2	4
One VUS in an AR PD gene	319	319
One VUS in an AD PD gene	586	586
One VUS in an AR PD gene + one VUS in an AD PD gene	24	48
Two VUSs in the same AD PD gene	15	30
Two VUSs in the same AD PD gene + one single heterozygous P/LP variant in an AR PD gene	1	3
Two VUSs in the same AD PD gene + one VUS in an AR PD gene	1	3
Two VUSs in the same AR PD gene	11	22
Two VUSs in the same AR PD gene + one VUS in an AD PD gene	1	3
Two VUSs in two different AD PD genes	5	10
Two VUSs in two different AR PD genes	4	8
Sum:	1252	1344

Unless stated otherwise (e.g., homozygous), a gene name (or AD PD or AR PD) denotes a single heterozygous pathogenic or likely pathogenic variant in a gene other than GBA. GBA denotes a single heterozygous risk factor.
PDGT: Parkinson's disease-relevant genetic testing;
AD PD gene: autosomal-dominant Parkinson's disease-related gene (LRRK2, GBA1, SNCA, VPS35, CHCHD2)
AR PD gene: autosomal-recessive Parkinson's disease-related gene (PRKN, PINK1, PARK7)

Supplementary Table 10. ROPAD study participants who received a positive Parkinson's disease-relevant genetic test (PDGT): Overview based on the family history status

Family history	All ROPAD participants	Number (and percentage) of patients with variants in respective genes						
		LRRK2		*GBA1*				*PRKN*
		All variants[a]	p.G2019S	All variants[a]	GD-relevant variants	p.E365K	p.T408M	All variants[a]
Positive	3394 (100%)	171 (5.04%)	142 (4.18%)	419 (12.35%)	239 (7.04%)	100 (2.95%)	99 (2.92%)	57 (1.68%)
Negative	8779 (100%)	207 (2.36%)	166 (1.89%)	868 (9.89%)	425 (4.84%)	271 (3.09%)	203 (2.31%)	63 (0.72%)

GD: Gaucher's disease; p.G2019S: p.Gly2019Ser; p.E365K: p.Glu365Lys; p.T408M: p.Thr408Met

[a]The "All variants" columns contain numbers of patients with a positive Parkinson's disease-relevant genetic test (PDGT) and with a positive or negative family history, respectively, while those with an unknown family history status were omitted. Therefore, these numbers do not amount to numbers of patients per the respective gene in Table 2A (e.g., for *LRRK2*: 171+207<368+23). In addition, some patients carried more than one variant in the same gene (Supplementary Table 4), and thus e.g., a sum of the numbers of patients with a positive family history and GD-relevant (n=239), p.E365K (n=100), and p.T408M (n=99) variants is higher than the number of patients with a positive family history and a positive PDGT based on *GBA1* variants (n=419).

Supplementary Table 11. Pair-wise statistical comparisons between various patient subgroups

Group	Variable	*P* value[a]	*P* value adjusted for sex and family history	Graphically represented in
Idiopathic vs. PDGT-positive	Age at onset	**8.94E-34**	**4.42E-37**	Fig. 3C
	Age at diagnosis	**3.28E-36**	**5.06E-39**	Fig. 3D
	Age at enrolment	**2.45E-33**	**8.75E-35**	Fig. 3E
	Sex	**1.12E-04**	-	Fig. 3A
	Fraction of participants with a FH+	**5.90E-18**	-	Fig. 3B
Idiopathic vs. *GBA1*	Age at onset	**2.64E-12**	**3.56E-12**	Fig. 4C
	Age at diagnosis	**4.09E-13**	**8.73E-13**	Fig. 4D
	Age at enrolment	**2.74E-16**	**9.96E-16**	Fig. 4E
	Sex	2.00E-01	-	Fig. 4A
	Fraction of participants with a FH+	4.98E-04	-	Fig. 4B
Idiopathic vs. *LRRK2*	Age at onset	2.29E-01	3.73E-01	Fig. 4C
	Age at diagnosis	1.14E-01	2.07E-01	Fig. 4D
	Age at enrolment	7.54E-01	7.23E-01	Fig. 4E
	Sex	**2.15E-04**	-	Fig. 4A
	Fraction of participants with a FH+	**1.38E-10**	-	Fig. 4B
Idiopathic vs. *PRKN/PINK1/PARK7*	Age at onset	**1.26E-56**	**1.58E-100**	Fig. 4C
	Age at diagnosis	**6.02E-55**	**7.59E-93**	Fig. 4D
	Age at enrolment	**1.81E-32**	**4.04E-51**	Fig. 4E
	Sex	1.59E-01	-	Fig. 4A
	Fraction of participants with a FH+	**1.16E-07**	-	Fig. 4B
Idiopathic vs. *SNCA*	Age at onset	**3.29E-04**	**3.24E-05**	Fig. 4C
	Age at diagnosis	**2.79E-04**	**1.43E-05**	Fig. 4D
	Age at enrolment	**7.76E-06**	**3.66E-09**	Fig. 4E
	Sex	9.99E-01	-	Fig. 4A
	Fraction of participants with a FH+	4.44E-04	-	Fig. 4B
GBA1 vs. *LRRK2*	Age at onset	6.52E-03	3.27E-03	Fig. 4C
	Age at diagnosis	1.17E-02	5.23E-03	Fig. 4D
	Age at enrolment	**1.01E-04**	**9.14E-05**	Fig. 4E
	Sex	1.10E-02	-	Fig. 4A
	Fraction of participants with a FH+	**3.29E-04**	-	Fig. 4B

PDGT: Parkinson's disease-relevant genetic test; FH+: positive family history;
[a]Statistically significant *P* values (upon correction for multiple testing) are shown in bold.

Supplementary Table 11. Continued

Group	Variable	*P* value[a]	*P* value adjusted for sex and family history	Graphically represented in
GBA1 vs. *PRKN/PINK1/PARK7*	Age at onset	**1.73E-44**	**3.18E-58**	Fig. 4C
	Age at diagnosis	**5.54E-42**	**6.72E-55**	Fig. 4D
	Age at enrolment	**2.50E-19**	**2.49E-24**	Fig. 4E
	Sex	4.13E-01	-	Fig. 4A
	Fraction of participants with a FH+	6.28E-04	-	Fig. 4B
GBA1 vs. *SNCA*	Age at onset	1.62E-02	5.15E-03	Fig. 4C
	Age at diagnosis	9.76E-03	2.46E-03	Fig. 4D
	Age at enrolment	8.25E-04	**3.25E-05**	Fig. 4E
	Sex	8.32E-01	-	Fig. 4A
	Fraction of participants with a FH+	6.95E-03	-	Fig. 4B
LRRK2 vs. *PRKN/PINK1/PARK7*	Age at onset	**1.29E-40**	**2.30E-52**	Fig. 4C
	Age at diagnosis	**4.91E-39**	**7.58E-50**	Fig. 4D
	Age at enrolment	**2.23E-22**	**7.22E-26**	Fig. 4E
	Sex	4.67E-01	-	Fig. 4A
	Fraction of participants with a FH+	5.37E-01	-	Fig. 4B
LRRK2 vs. *SNCA*	Age at onset	9.90E-04	**1.65E-04**	Fig. 4C
	Age at diagnosis	9.10E-04	**7.34E-05**	Fig. 4D
	Age at enrolment	**3.10E-05**	**3.15E-07**	Fig. 4E
	Sex	3.23E-01	-	Fig. 4A
	Fraction of participants with a FH+	1.73E-01	-	Fig. 4B
PRKN/PINK1/PARK7 vs. *SNCA*	Age at onset	**1.24E-04**	**2.32E-05**	Fig. 4C
	Age at diagnosis	5.06E-04	**1.25E-04**	Fig. 4D
	Age at enrolment	5.73E-01	8.38E-01	Fig. 4E
	Sex	5.87E-01	-	Fig. 4A
	Fraction of participants with a FH+	3.47E-01	-	Fig. 4B
Idiopathic vs. *LRRK2*	Age at onset	2.33E-01	4.24E-01	Supp. Fig. 8C
	Age at diagnosis	9.13E-02	1.87E-01	Supp. Fig. 8D
	Age at enrolment	8.77E-01	8.87E-01	Supp. Fig. 8E
	Sex	**1.39E-05**	-	Supp. Fig. 8A
	Fraction of participants with a FH+	**1.09E-14**	**-**	Supp. Fig. 8B

PDGT: Parkinson's disease-relevant genetic test; FH+: positive family history;
[a]Statistically significant *P* values (upon correction for multiple testing) are shown in bold.

Supplementary Table 11. Continued

Group	Variable	*P* value[a]	*P* value adjusted for sex and family history	Graphically represented in
Idiopathic vs. p.E365K *GBA1*	Age at onset	3.66E-03	2.46E-03	Supp. Fig. 8C
	Age at diagnosis	2.30E-02	2.88E-02	Supp. Fig. 8D
	Age at enrolment	6.56E-02	1.09E-01	Supp. Fig. 8E
	Sex	3.83E-01	-	Supp. Fig. 8A
	Fraction of participants with a FH+	8.19E-01	-	Supp. Fig. 8B
Idiopathic vs. p.T408M *GBA1*	Age at onset	1.20E-02	4.41E-03	Supp. Fig. 8C
	Age at diagnosis	7.16E-03	2.50E-03	Supp. Fig. 8D
	Age at enrolment	**1.08E-04**	**1.79E-04**	Supp. Fig. 8E
	Sex	9.08E-02	-	Supp. Fig. 8A
	Fraction of participants with a FH+	4.45E-02	-	Supp. Fig. 8B
Idiopathic vs. Gaucher's disease-relevant *GBA1* variants	Age at onset	**4.51E-17**	**1.56E-14**	Supp. Fig. 8C
	Age at diagnosis	**3.25E-20**	**1.03E-17**	Supp. Fig. 8D
	Age at enrolment	**1.03E-24**	**1.76E-23**	Supp. Fig. 8E
	Sex	2.41E-01	-	Supp. Fig. 8A
	Fraction of participants with a FH+	**2.11E-06**	-	Supp. Fig. 8B
Idiopathic vs. *LRRK2+GBA*	Age at onset	2.28E-01	2.90E-01	Supp. Fig. 8C
	Age at diagnosis	3.00E-01	3.50E-01	Supp. Fig. 8D
	Age at enrolment	8.76E-01	7.53E-01	Supp. Fig. 8E
	Sex	3.13E-02	-	Supp. Fig. 8A
	Fraction of participants with a FH+	1.04E-01	-	Supp. Fig. 8B
LRRK2 vs. p.E365K *GBA1*	Age at onset	2.01E-01	5.59E-02	Supp. Fig. 8C
	Age at diagnosis	6.86E-01	2.96E-01	Supp. Fig. 8D
	Age at enrolment	1.62E-01	1.28E-01	Supp. Fig. 8E
	Sex	**2.50E-04**	-	Supp. Fig. 8A
	Fraction of participants with a FH+	**1.54E-06**	-	Supp. Fig. 8B
LRRK2 vs. p.T408M *GBA1*	Age at onset	2.45E-01	8.43E-02	Supp. Fig. 8C
	Age at diagnosis	3.34E-01	1.23E-01	Supp. Fig. 8D
	Age at enrolment	2.61E-03	4.54E-03	Supp. Fig. 8E
	Sex	1.34E-01	-	Supp. Fig. 8A
	Fraction of participants with a FH+	1.12E-03	-	Supp. Fig. 8B

PDGT: Parkinson's disease-relevant genetic test; FH+: positive family history;
[a]Statistically significant p values (upon correction for multiple testing) are shown in bold.

Supplementary Table 11. Continued

Group	Variable	*P* value[a]	*P* value adjusted for sex and family history	Graphically represented in
LRRK2 vs. Gaucher's disease-relevant *GBA1* variants	Age at onset	**2.36E-06**	**1.47E-05**	Supp. Fig. 8C
	Age at diagnosis	**1.01E-06**	**6.61E-06**	Supp. Fig. 8D
	Age at enrolment	**5.11E-11**	**1.03E-09**	Supp. Fig. 8E
	Sex	8.07E-03	-	Supp. Fig. 8A
	Fraction of participants with a positive FH+	4.03E-03	-	Supp. Fig. 8B
LRRK2 vs. *LRRK2+GBA*	Age at onset	3.56E-01	4.27E-01	Supp. Fig. 8C
	Age at diagnosis	5.40E-01	6.08E-01	Supp. Fig. 8D
	Age at enrolment	8.52E-01	8.02E-01	Supp. Fig. 8E
	Sex	3.48E-01	-	Supp. Fig. 8A
	Fraction of participants with a FH+	1.00E+00	-	Supp. Fig. 8B
p.E365K vs. p.T408M *GBA1*	Age at onset	8.96E-01	9.62E-01	Supp. Fig. 8C
	Age at diagnosis	6.53E-01	5.21E-01	Supp. Fig. 8D
	Age at enrolment	6.66E-02	1.14E-01	Supp. Fig. 8E
	Sex	6.14E-02	-	Supp. Fig. 8A
	Fraction of participants with a FH+	2.20E-01	-	Supp. Fig. 8B
p.E365K vs. Gaucher's disease-relevant GBA1 variants	Age at onset	8.30E-04	2.36E-02	Supp. Fig. 8C
	Age at diagnosis	**9.87E-06**	**3.12E-04**	Supp. Fig. 8D
	Age at enrolment	**9.29E-08**	**2.31E-06**	Supp. Fig. 8E
	Sex	1.48E-01	-	Supp. Fig. 8A
	Fraction of participants with a FH+	1.18E-02	-	Supp. Fig. 8B
p.E365K *GBA1* vs. *LRRK2+GBA*	Age at onset	5.74E-01	9.63E-01	Supp. Fig. 8C
	Age at diagnosis	6.31E-01	9.41E-01	Supp. Fig. 8D
	Age at enrolment	7.50E-01	5.82E-01	Supp. Fig. 8E
	Sex	2.00E-02	-	Supp. Fig. 8A
	Fraction of participants with a FH+	1.48E-01	-	Supp. Fig. 8B
p.T408M vs. Gaucher's disease-relevant *GBA1* variants	Age at onset	2.56E-03	3.55E-02	Supp. Fig. 8C
	Age at diagnosis	8.53E-04	1.27E-02	Supp. Fig. 8D
	Age at enrolment	1.68E-03	7.85E-03	Supp. Fig. 8E
	Sex	4.99E-01	-	Supp. Fig. 8A
	Fraction of participants with a FH+	3.56E-01	-	Supp. Fig. 8B

PDGT: Parkinson's disease-relevant genetic test; FH+: positive family history;
[a]Statistically significant *P* values (upon correction for multiple testing) are shown in bold.

Supplementary Table 11. Continued

Group	Variable	*P* value[a]	*P* value adjusted for sex and family history	Graphically represented in
p.T408M *GBA1* vs. *LRRK2+GBA*	Age at onset	5.58E-01	9.13E-01	Supp. Fig. 8C
	Age at diagnosis	7.37E-01	9.47E-01	Supp. Fig. 8D
	Age at enrolment	3.80E-01	3.95E-01	Supp. Fig. 8E
	Sex	1.29E-01	-	Supp. Fig. 8A
	Fraction of participants with a FH+	3.76E-01	-	Supp. Fig. 8B
Gaucher's disease-relevant *GBA1* variants vs. *LRRK2+GBA1*	Age at onset	6.53E-01	5.76E-01	Supp. Fig. 8C
	Age at diagnosis	3.73E-01	3.93E-01	Supp. Fig. 8D
	Age at enrolment	7.19E-02	9.60E-02	Supp. Fig. 8E
	Sex	6.67E-02	-	Supp. Fig. 8A
	Fraction of participants with a FH+	5.83E-01	-	Supp. Fig. 8B

PDGT: Parkinson's disease-relevant genetic test; FH+: positive family history;
[a]Statistically significant *P* values (upon correction for multiple testing) are shown in bold.

Supplementary Table 12. Results for logistic regression prediction the probability of a positive Parkinson's disease-relevant genetic test based on age at onset, sex, and family history

Variable	Estimate (beta)	Standard Error	Odds ratio	*P* value
Intercept	0.0132	0.1272	1.0133	9.18E-01
Age at onset	-0.0272	0.0022	0.9732	1.04E-35
Sex (female)	0.1986	0.0550	1.2197	3.02E-04
Positive family history	0.4390	0.0570	1.5511	1.41E-14

Supplementary Figures

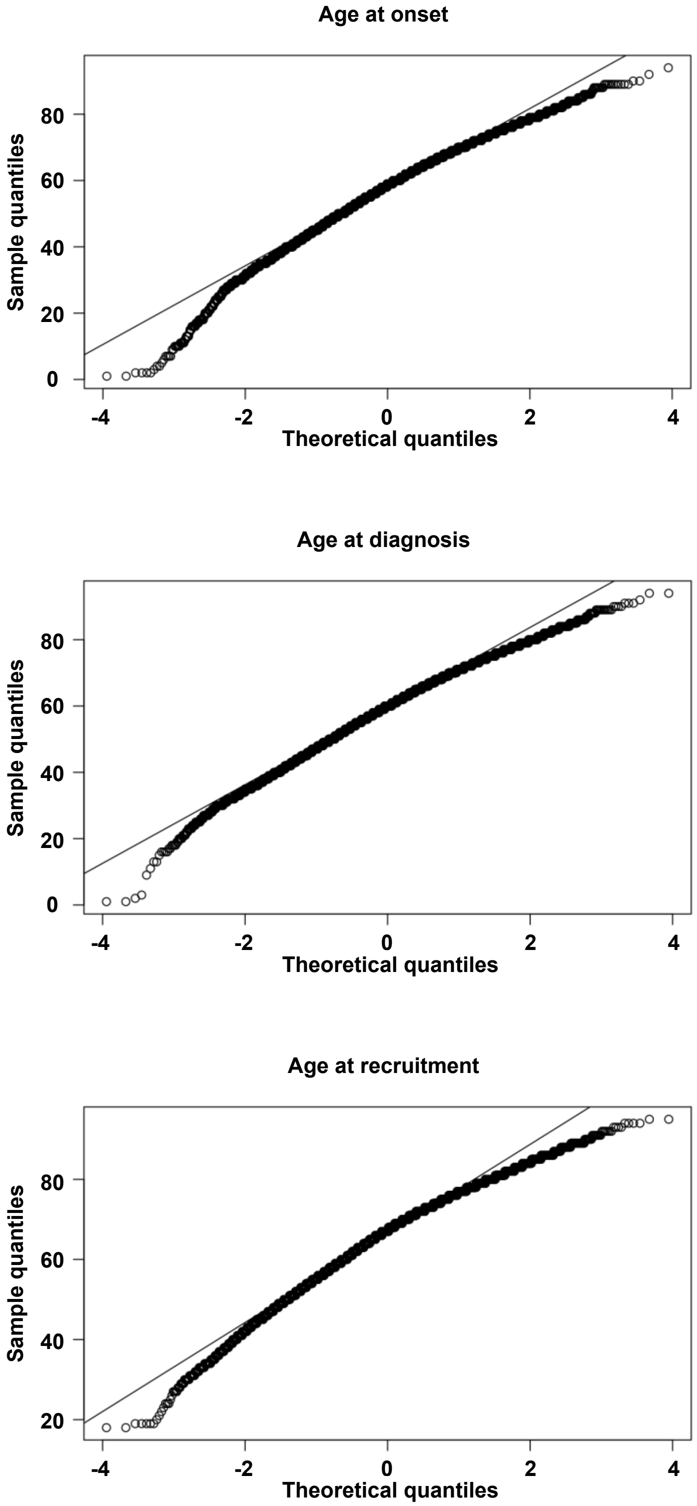

Supplementary Figure 1. Assessing data normality of the age at onset, age at clinical diagnosis, and age at recruitment in the investigated ROPAD study patients group using Q-Q plots. All continuous variables showed deviations from a normal distribution.

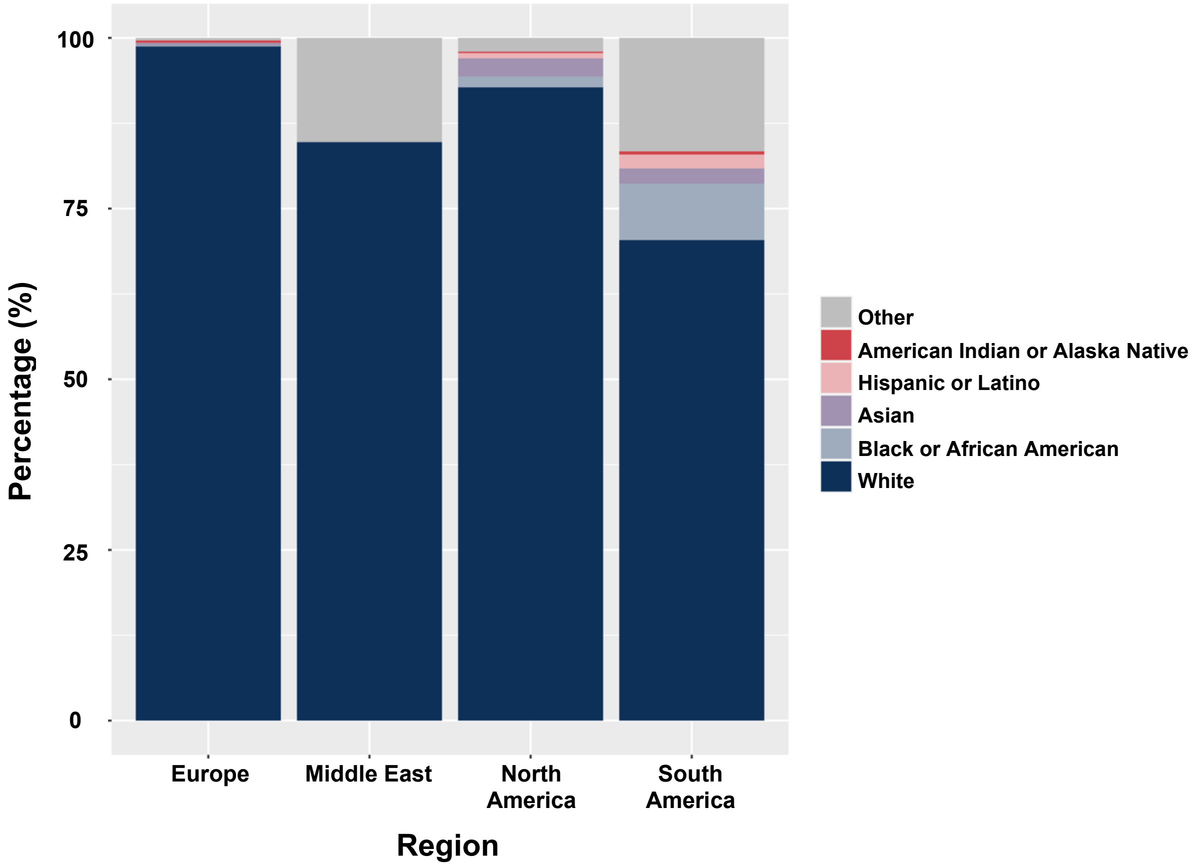

Supplementary Figure 2. Race/ethnicity of the 12580 investigated ROPAD study participants, as reported in the electronic case report form (eCRF).

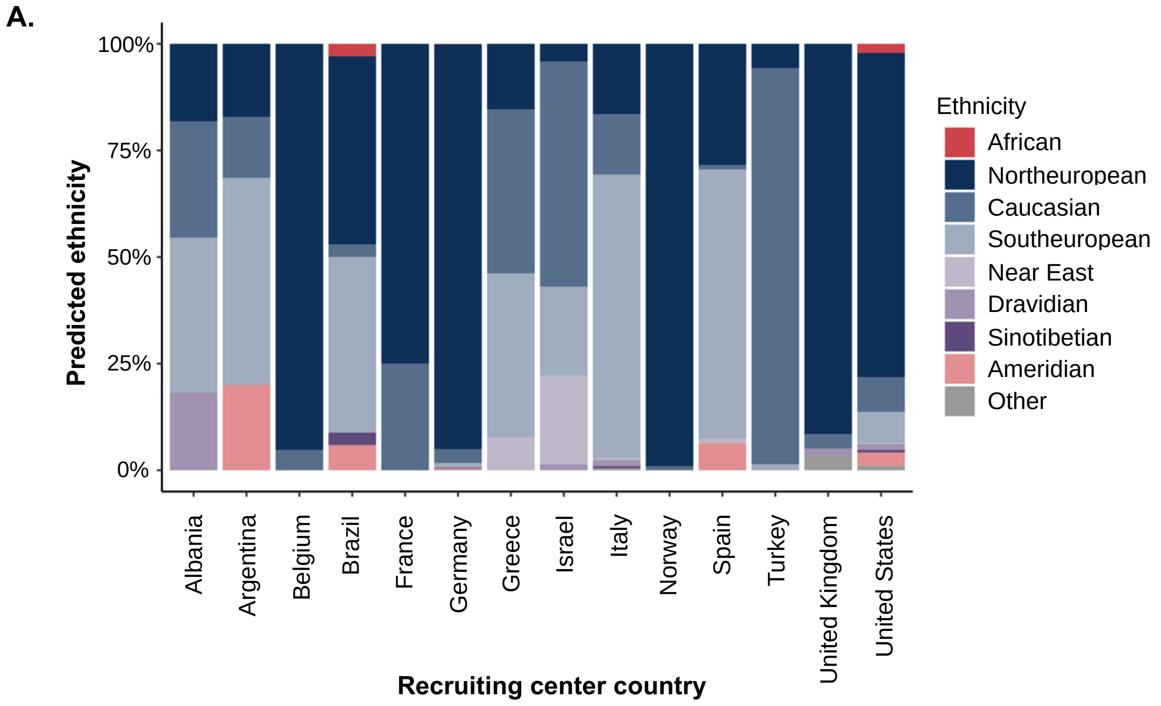

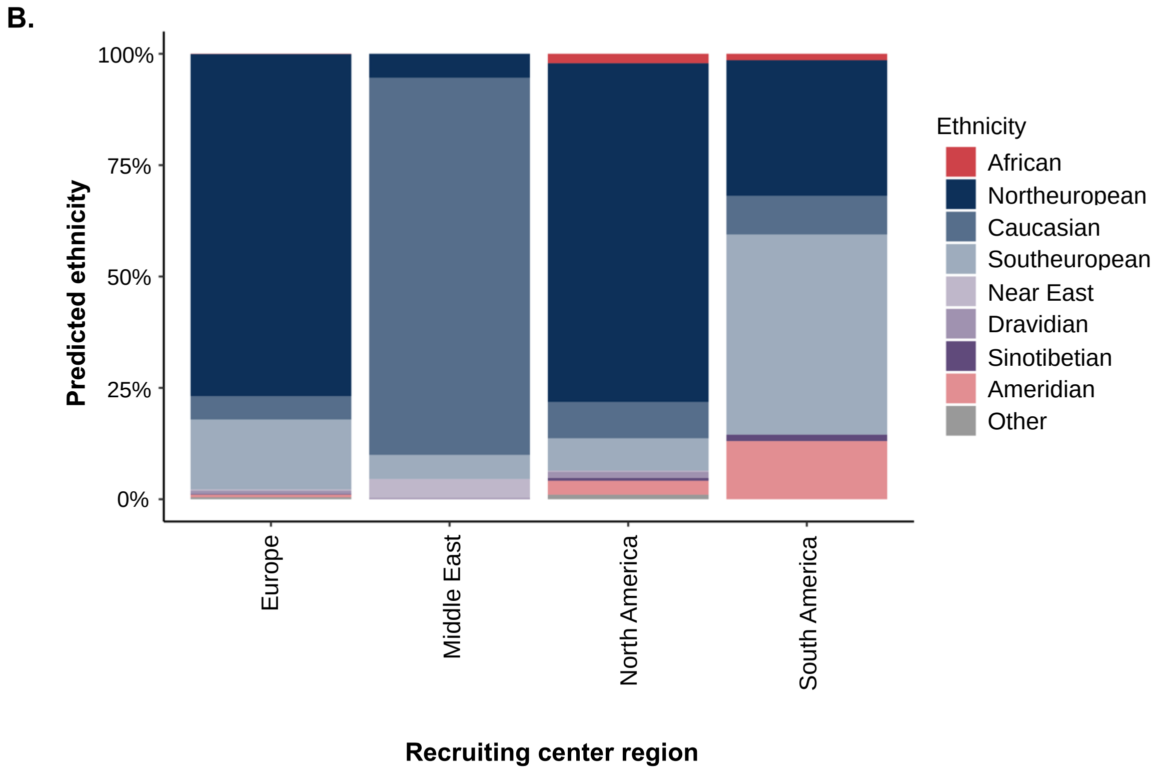

Supplementary Figure 3. Ethnicity predicted based on whole-genome sequencing data from 2587 ROPAD study participants grouped per country (A) or region (B) they were recruited in.

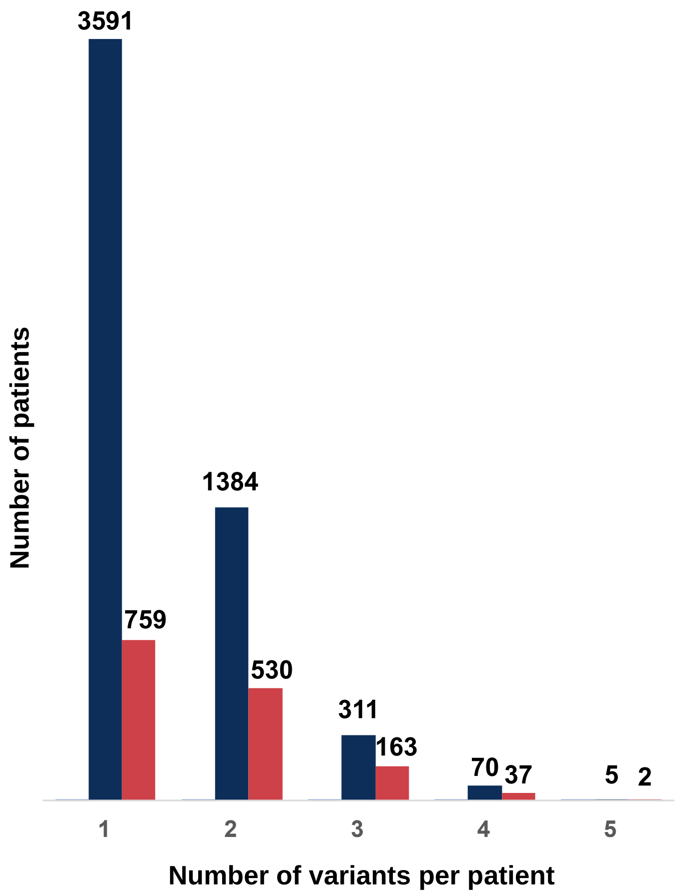

Supplementary Figure 4. Distribution of the number of pathogenic and likely pathogenic variants, variants of uncertain significance, and risk factors (the latter applies to *GBA1*) detected by panel sequencing per patient. All patients (n=5361 with 7597 variants) (dark blue) and patients who received a Parkinson's disease (PD)-relevant genetic test (PDGT) report (n=1491, with 2466 variants) (red).

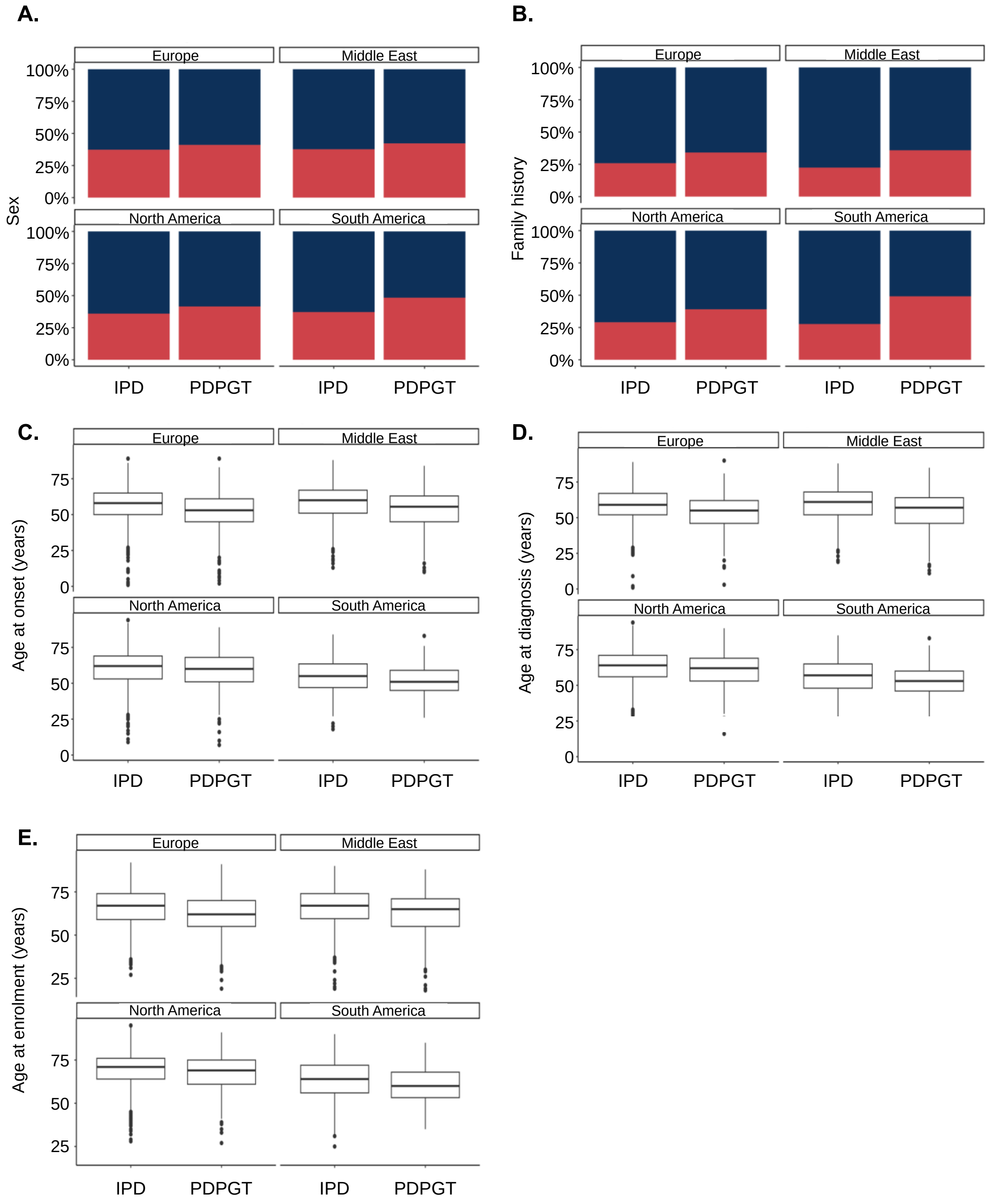

Supplementary Figure 5. Comparison of demographic and age-related variables between idiopathic PD (IPD) patients and those with a positive PD-relevant genetic test (PDPGT) stratified by the geographic region of the recruitment center. (A) Sex (blue: male, red: female), (B) Fractions of patients with positive family history (red: positive family history, blue: negative family history), (C) Age at onset, (D) Age at diagnosis, (E) Age at enrolment.

A.

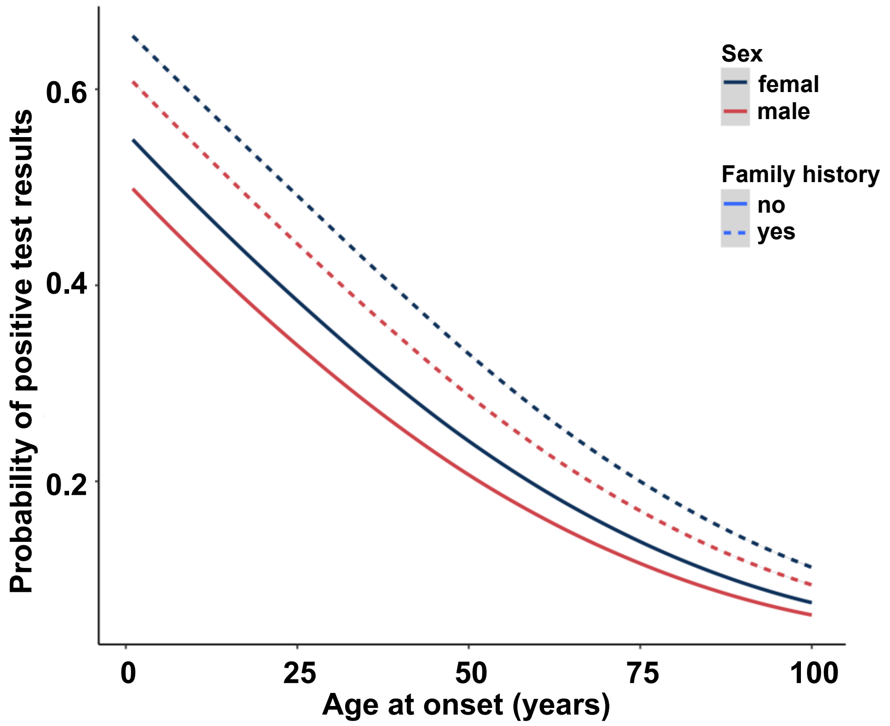

B.

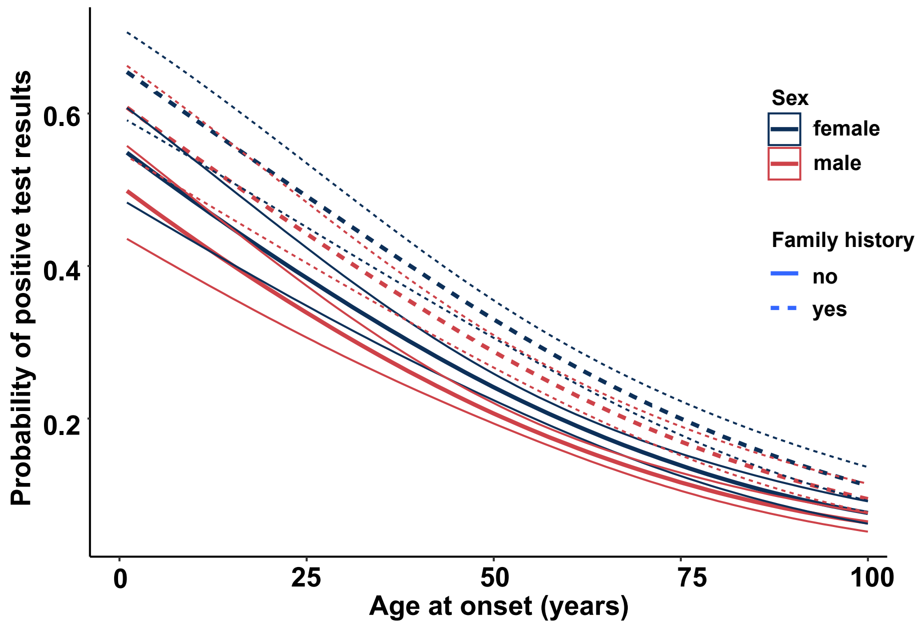

Supplementary Figure 6. Predicted probability to get a positive PDGT result, based on a logistic regression model including AAO, sex, and family history as predictor variables without (A) and with (B) confidence lines.

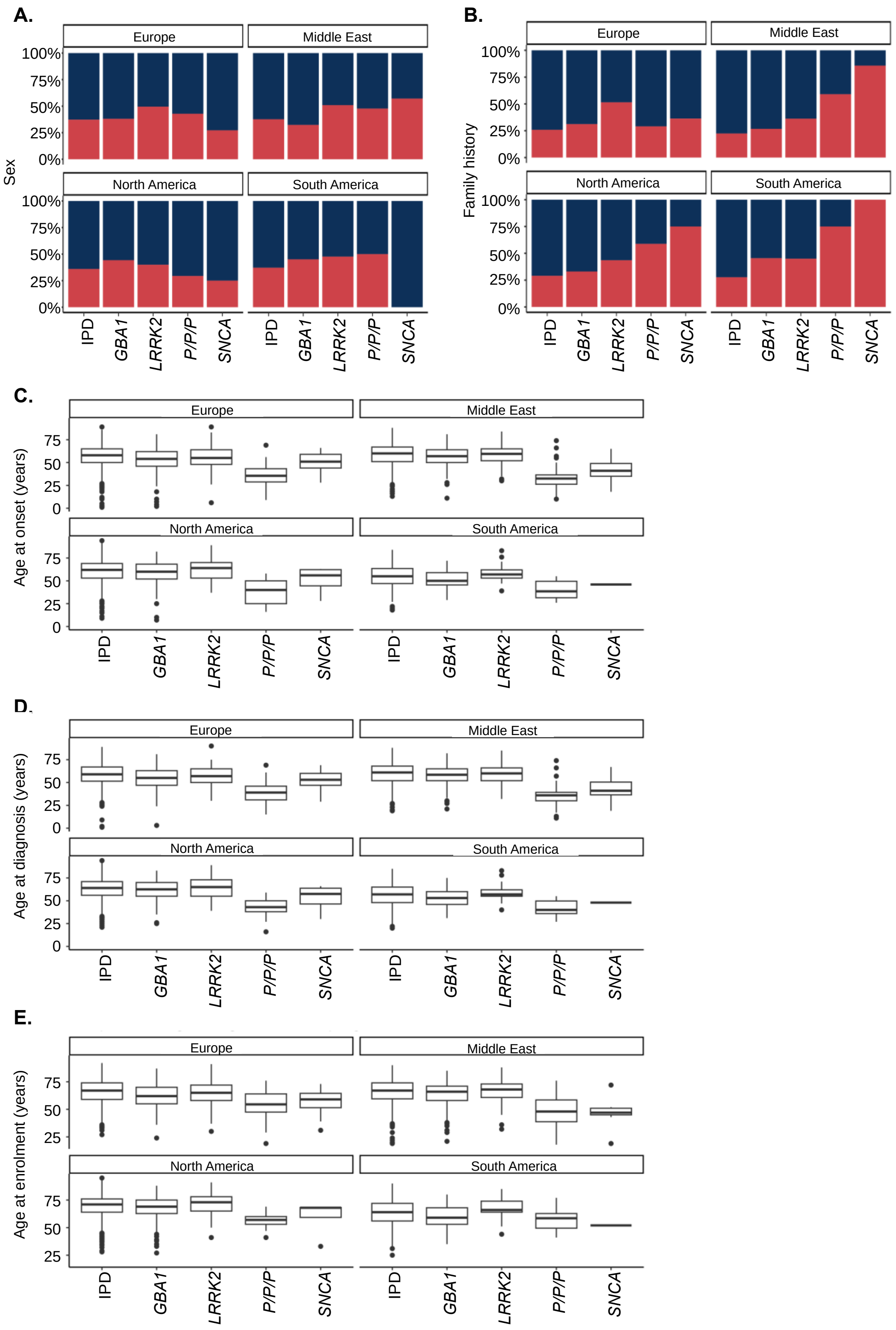

Supplementary Figure 7. Comparison of demographic and age-related variables between idiopathic PD (IPD) patients and four different genetic patient subgroups (positive PD-relevant genetic test (PDGT) based on: *GBA1, LRRK2, PRKN/PINK1/PARK7 (P/P/P),* or *SNCA* variants stratified by the geographic region of the recruitment center. (A) Sex (blue: male, red: female), (B) Fractions of patients with positive family history (red: positive family history, blue: negative family history), (C) Age at onset, (D) Age at diagnosis, (E) Age at enrolment.

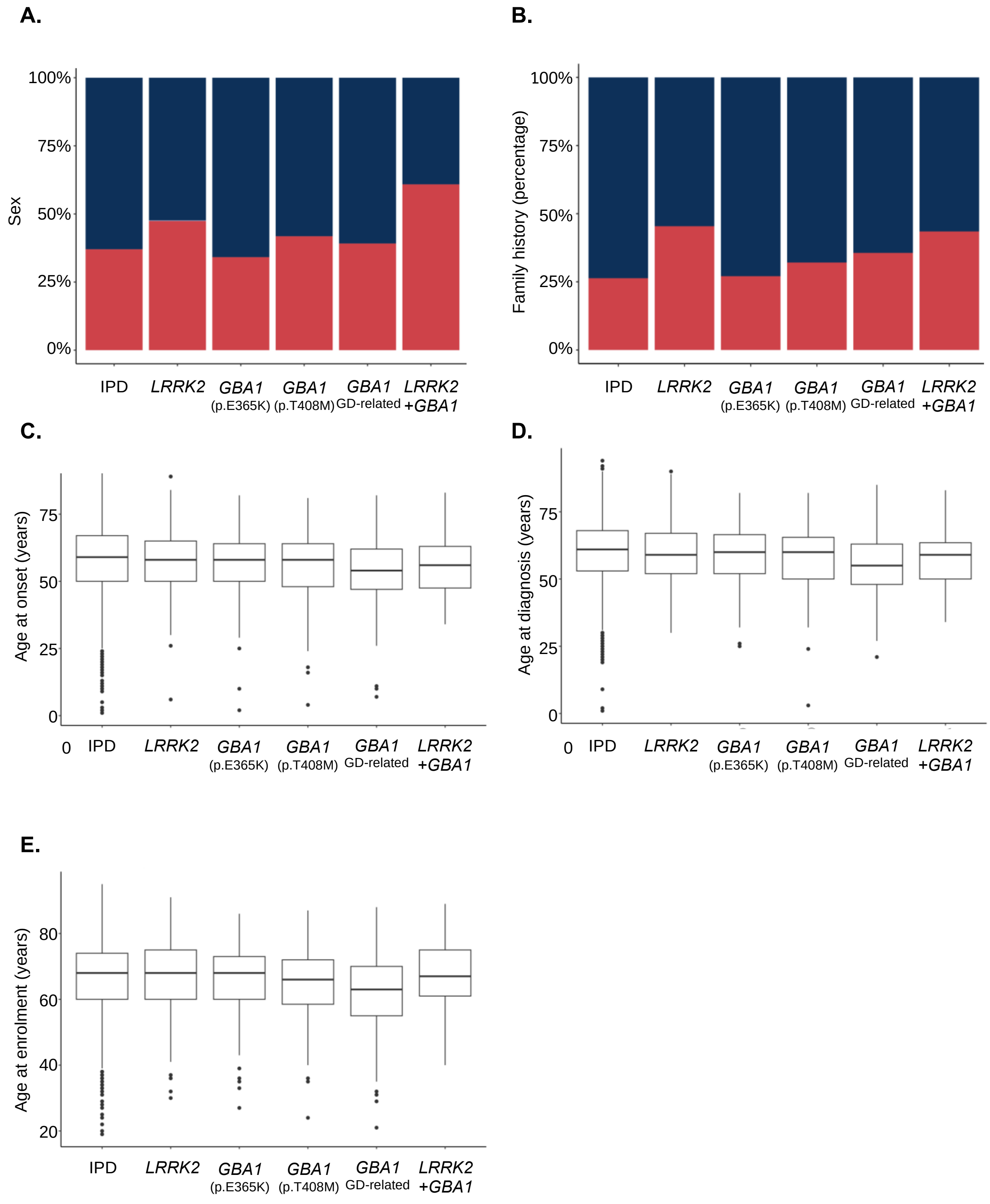

Supplementary Figure 8. Comparison of demographic and age-related variables between idiopathic PD patients and patients with positive PD-relevant genetic testing (PDGT) reports based on: LRRK2, GBA1 subtypes (p.Glu365Lys (p.E365K), p.Thr408Met (p.T408M), and Gaucher disease-relevant GBA1 variants), and LRRK2+GBA1 (A) Sex (blue: male, red: female), (B) Fractions of patients with positive family history (red: positive family history, blue: negative family history), (C) Age at onset, (D) Age at diagnosis, (E) Age at enrolment.

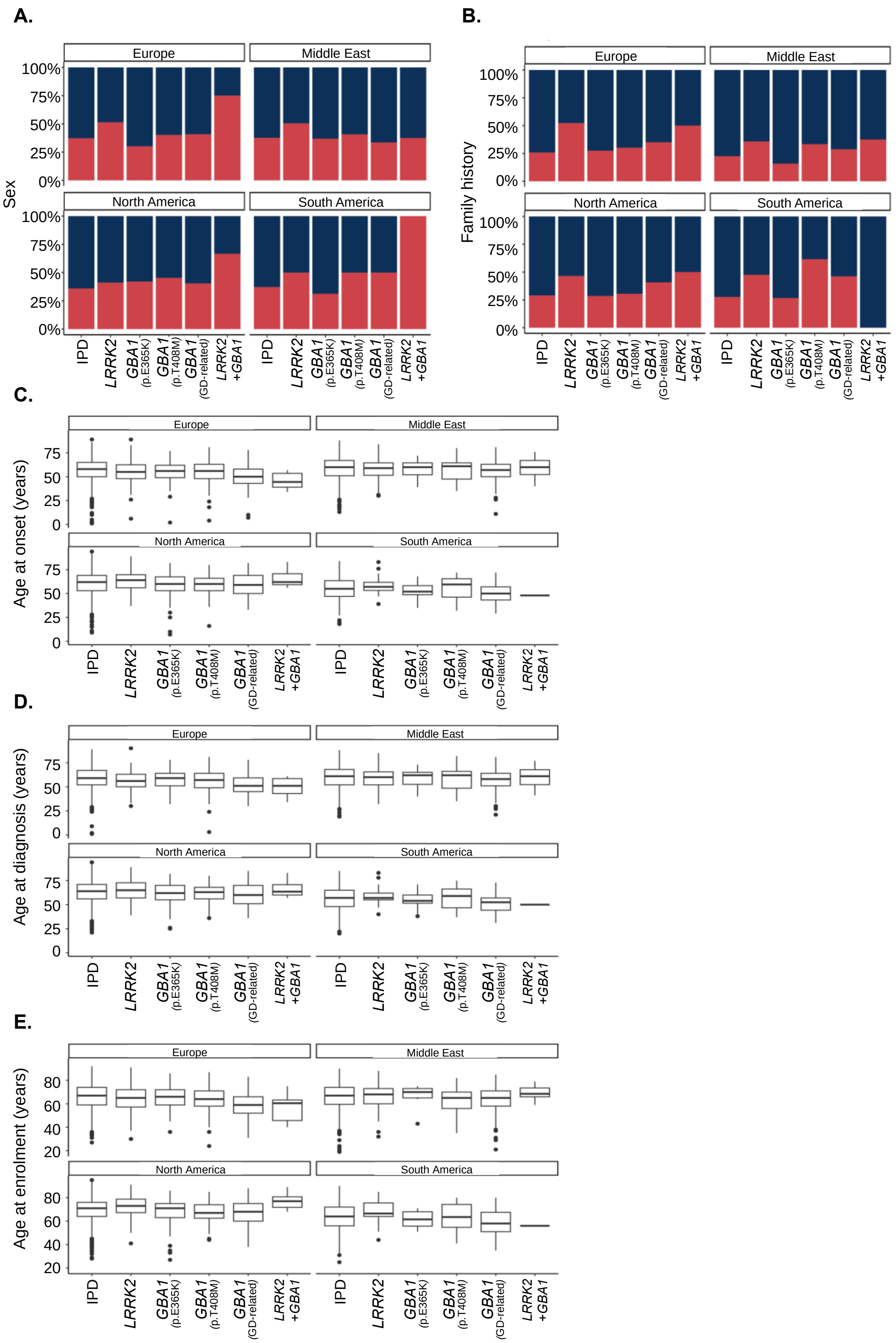

Supplementary Figure 9. Comparison of demographic and age-related variables between idiopathic PD (IPD) patients and patients with positive PD-relevant genetic testi (PDGT) based on: *LRRK2, GBA1* subtypes (p.Glu365Lys (p.E365K), p.Thr408Met (p.T408M), and Gaucher disease-relevant *GBA1* variants), and *LRRK2+GBA1* stratified by the geographic region of the recruitment center. (A) Sex (blue: male, red: female), (B) Fractions of patients with positive family history (red: positive family history, blue: negative family history), (C) Age at onset, (D) Age at diagnosis, (E) Age at enrolment.

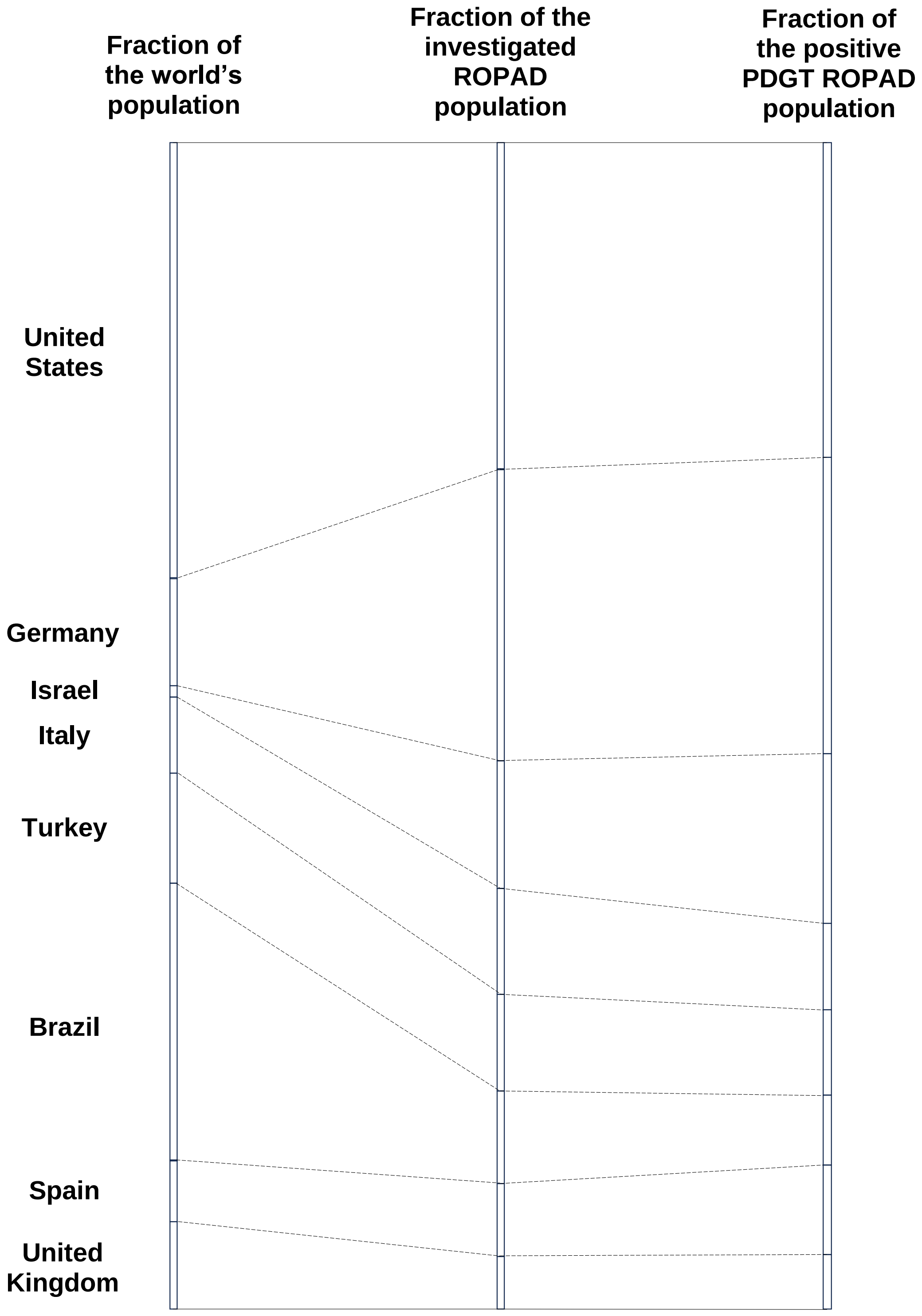

Supplementary Figure 10. Eight countries with over 500 recruited investigated PD patients and their fractions in terms of number of inhabitants (left), number of investigated ROPAD study patients (middle), and number of individuals with a positive Parkinson's disease-relevant genetic test (PDGT) (right).
